# Dissecting the Arginine and Lysine Biosynthetic Pathways and Their Relationship in Haloarchaeon Natrinema gari J7-2 via Endogenous CRISPR-Cas System-Based Genome Editing

**DOI:** 10.1128/spectrum.00288-23

**Published:** 2023-06-22

**Authors:** Yi Wu, Jia Zhang, Bingxue Wang, Yanyan Zhang, Huai Li, Yang Liu, Jing Yin, Dan He, Hongyi Luo, Fei Gan, Bing Tang, Xiao-Feng Tang

**Affiliations:** a Hubei Key Laboratory of Cell Homeostasis, College of Life Sciences, Wuhan University, Wuhan, China; b State Key Laboratory of Virology, College of Life Sciences, Wuhan University, Wuhan, China; c Cooperative Innovation Center of Industrial Fermentation (Ministry of Education & Hubei Province), Wuhan, China; Huazhong Agricultural University

**Keywords:** haloarchaea, CRISPR, genome editing, arginine biosynthesis, lysine biosynthesis, diaminopimelate pathway, α-aminoadipate pathway

## Abstract

The evolutionary relationship between arginine and lysine biosynthetic pathways has been well established in bacteria and hyperthermophilic archaea but remains largely unknown in haloarchaea. Here, the endogenous CRISPR-Cas system was harnessed to edit arginine and lysine biosynthesis-related genes in the haloarchaeon Natrinema gari J7-2. The Δ*argW*, Δ*argX*, Δ*argB*, and Δ*argD* mutant strains display an arginine auxotrophic phenotype, while the Δ*dapB* mutant shows a lysine auxotrophic phenotype, suggesting that strain J7-2 utilizes the ArgW-mediated pathway and the diaminopimelate (DAP) pathway to synthesize arginine and lysine, respectively. Unlike the ArgD in Escherichia coli acting as a bifunctional aminotransferase in both the arginine biosynthesis pathway and the DAP pathway, the ArgD in strain J7-2 participates only in arginine biosynthesis. Meanwhile, in strain J7-2, the function of *argB* cannot be compensated for by its evolutionary counterpart *ask* in the DAP pathway. Moreover, strain J7-2 cannot utilize α-aminoadipate (AAA) to synthesize lysine via the ArgW-mediated pathway, in contrast to hyperthermophilic archaea that employ a bifunctional LysW-mediated pathway to synthesize arginine (or ornithine) and lysine from glutamate and AAA, respectively. Additionally, the replacement of a 5-amino-acid signature motif responsible for substrate specificity of strain J7-2 ArgX with that of its hyperthermophilic archaeal homologs cannot endow the Δ*dapB* mutant with the ability to biosynthesize lysine from AAA. The *in vitro* analysis shows that strain J7-2 ArgX acts on glutamate rather than AAA. These results suggest that the arginine and lysine biosynthetic pathways of strain J7-2 are highly specialized during evolution.

**IMPORTANCE** Due to their roles in amino acid metabolism and close evolutionary relationship, arginine and lysine biosynthetic pathways represent interesting models for probing functional specialization of metabolic routes. The current knowledge with respect to arginine and lysine biosynthesis is limited for haloarchaea compared to that for bacteria and hyperthermophilic archaea. Our results demonstrate that the haloarchaeon Natrinema gari J7-2 employs the ArgW-mediated pathway and the DAP pathway for arginine and lysine biosynthesis, respectively, and the two pathways are functionally independent of each other; meanwhile, ArgX is a key determinant of substrate specificity of the ArgW-mediated pathway in strain J7-2. This study provides new clues about haloarchaeal amino acid metabolism and confirms the convenience and efficiency of endogenous CRISPR-Cas system-based genome editing in haloarchaea.

## INTRODUCTION

**FIG 1:**
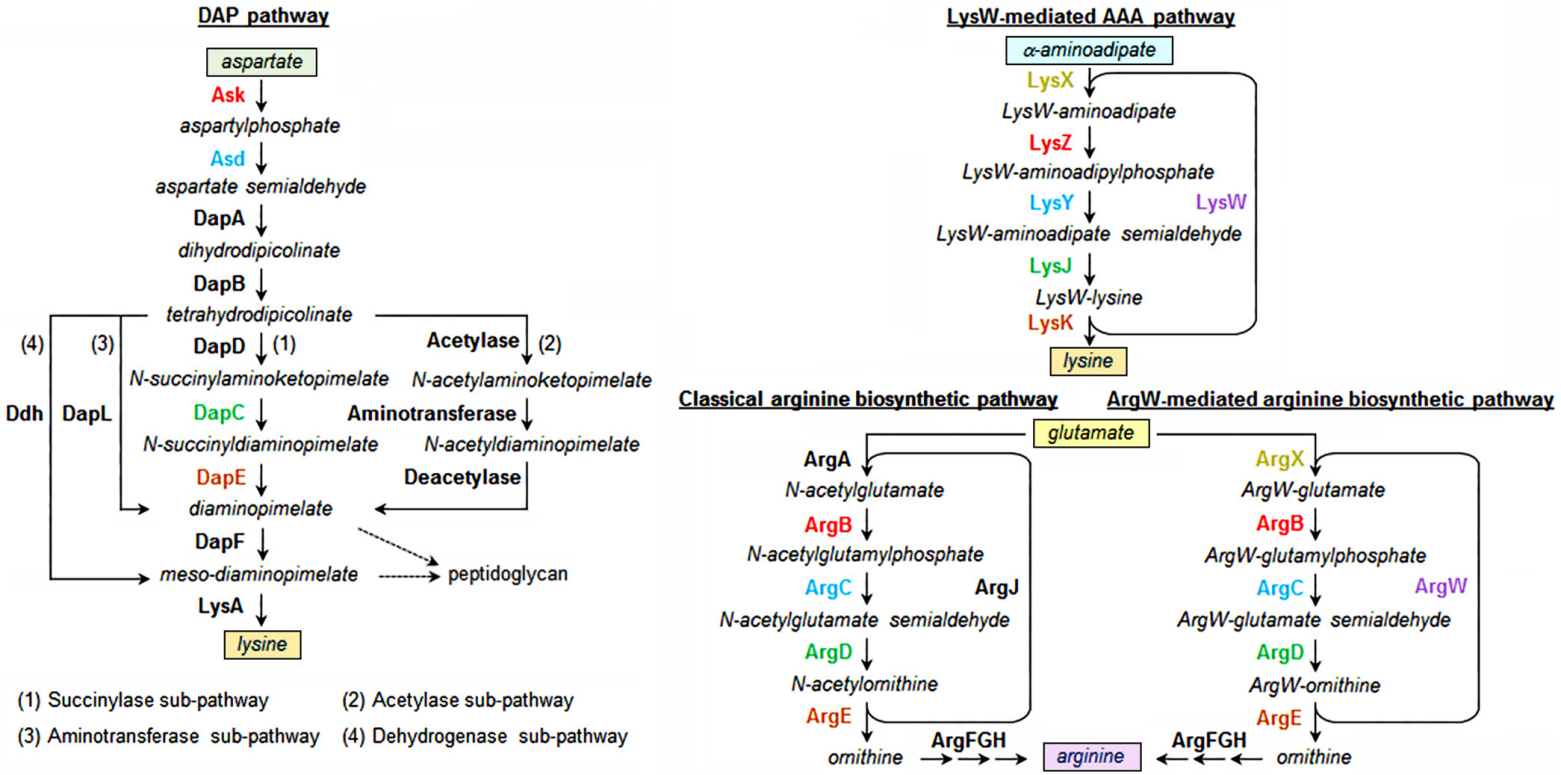
Lysine and arginine biosynthetic pathways in prokaryotes. The four subpathways of the DAP pathway are indicated (numbered 1 to 4). The proteins sharing the same color (cyan, red, blue, green, brown, or purple) in different pathways are homologs.

Prokaryotes synthesize arginine from glutamate, and lysine is synthesized from aspartate via the diaminopimelate (DAP) pathway or from α-aminoadipate (AAA) via the AAA pathway; the DAP pathway also plays a central role in peptidoglycan synthesis in Gram-negative bacteria (1–3). Depending on microbial species, the DAP pathway can be divided into four subpathways, namely, the succinylase, acetylase, aminotransferase, and dehydrogenase subpathways, which share the steps converting aspartate to tetrahydrodipicolinate but differ from each other in subsequent steps to form *meso*-DAP, the precursor of lysine (Fig. 1) (4). The thermophilic bacterium Thermus thermophilus possesses the classical arginine biosynthetic pathway (5); it lacks the DAP pathway but utilizes LysX to catalyze the covalent linkage of the amino group of AAA to the γ-carboxyl group of the C-terminal residue Glu^54^ of a carrier protein, LysW, for lysine biosynthesis, known as the LysW-mediated AAA pathway, involving enzymes similar to those of the arginine biosynthetic pathway (Fig. 1) (6). For archaea, methanogens employ the classical arginine biosynthetic pathway (7) and the aminotransferase subpathway (8, 9) for arginine and lysine biosynthesis, respectively, and *argG* of Methanococcus voltae could complement the *argG* mutation in an arginine auxotroph of Escherichia coli (10); hyperthermophilic archaea utilize the LysW-mediated AAA pathway and the ArgW (a LysW homolog)-mediated arginine biosynthetic pathway to synthesize lysine and arginine, respectively (Fig. 1) (11, 12).

It is generally assumed that early life-forms have small genomes encoding enzymes with broad specificities to interconnect different metabolic pathways, and the primitive enzymes will be evolved into multiple homologous enzymes with a narrowed substrate specificity due to gene duplication and specialization during the process of evolution (13). Several lines of evidence reveal the close evolutionary relationship and functional specialization of the biosynthetic pathways for arginine and lysine (1, 2). It has been shown that the enzymes Ask, Asd, DapC, and DapE of the DAP pathway, LysZ, LysY, LysJ, and LysK of the AAA pathway, and ArgB, ArgC, ArgD, and ArgE of the arginine biosynthetic pathway are homologs, respectively (Fig. 1) (1, 3). In Escherichia coli, the *N*-acetylornithine transaminase (ArgD) fulfills transamination reactions in both the arginine biosynthetic pathway and the DAP pathway for lysine (14), while in Corynebacterium glutamicum, two distinct enzymes (ArgD and DapC) catalyze the reactions in the two pathways, respectively (15). The close evolutionary relationship of the biosynthetic pathways of arginine and lysine is more evident in hyperthermophilic archaea. Sulfolobus acidocaldarius employs two homologous enzymes, ArgX and LysX, to catalyze the covalent linkage of glutamate and AAA with a common carrier protein, LysW, respectively, and the modified glutamate and AAA are then converted to arginine and lysine using a single set of enzymes with dual functions (11). Moreover, in Thermococcus kodakarensis both the modification of glutamate and AAA by LysW and subsequent reactions in the biosynthetic pathways for ornithine (precursor of arginine) and lysine are catalyzed by a single set of bifunctional enzymes, although this archaeon shows an arginine auxotrophic phenotype due to the lack of ArgG and ArgH required for conversion of ornithine to arginine (12).

In the case of haloarchaea, an isotopic labeling study shows that Haloarcula hispanica employs the DAP pathway to synthesize lysine (16); the mutagenesis of *dapD* in Natrinema gari J7-2 (previously *Natrinema* sp. strain J7-2) (17) generates a lysine auxotroph, implying that strain J7-2 employs the succinylase subpathway for lysine biosynthesis (Fig. 1) (18). Nevertheless, the gene *dapC* required for the succinylase subpathway (Fig. 1) is missing in the gene cluster of the DAP pathway in haloarchaea, and it is assumed that the function of *dapC* is fulfilled by *argD* of the arginine biosynthetic pathway as in E. coli (14, 19). Additionally, haloarchaeal genomes commonly possess a complete set of genes involved in ArgW-mediated arginine biosynthesis, including *argG*, *argH*, *argW*, *argX*, *argC*, *argB*, *argD*, *argE*, and *argF* (Fig. 1) (20, 21). The mutagenesis of *argB*, *argC*, *argD*, or *argH* of Haloferax volcanii or *Nnm. gari* J7-2 generates an arginine auxotroph (18, 21, 22). However, the functions of the “landmark” genes (*argW* and *argX*) of the ArgW-mediated arginine biosynthetic pathway of haloarchaea have not been confirmed experimentally, and it is unclear whether this pathway could also be used for lysine biosynthesis as in hyperthermophilic archaea (11, 12). It is of great interest to dissect the functions of genes involved in arginine and lysine biosynthesis of haloarchaea and their possible evolutionary relationships.

The CRISPR-Cas (clustered regularly interspaced short palindromic repeats with Cas) system is a unique RNA-guided acquired immune system in prokaryotes, and has been widely used in genome editing and gene function analysis in diverse organisms (23). However, heterologous CRISPR-Cas systems have the disadvantages of being toxic to some bacterial cells and low activity in certain hosts, particularly extremophiles (24, 25). An alternative strategy for genome editing is to employ endogenous CRISPR-Cas systems, which are encoded by most archaeal genomes and approximately half of bacterial genomes (26). Endogenous CRISPR-Cas system-based genome editing was first established in Streptococcus pneumoniae (27) and has been successfully applied in many bacteria and archaea (28–33). For haloarchaea, the use of endogenous type I-B CRISPR-Cas system for genome editing in Haloferax volcanii is hindered by its high tolerance to CRISPR-mediated self-targeting, likely because the high copy numbers of chromosome in this haloarchaeon mediate accurate genome repair via homologous recombination (34). In contrast, the native type I-B CRISPR-Cas system of the polyploid haloarchaeon *Har. hispanica* has been successfully harnessed for genome editing, highlighting the convenience and efficiency of endogenous CRISPR-Cas system-based genome editing in haloarchaea (35).

*Nnm. gari* J7-2 was isolated from a salt mine in China (36), and three extracellular subtilisin-like proteases (SptA, SptC, and SptE) of strain J7-2 have been characterized (37–40). Gene deletion analysis shows that the major extracellular protease SptA is not necessary for cell survival but facilitates the growth of strain J7-2 by degrading protein substrates into peptides and amino acids, which serve as nutrients for living cells (41). Meanwhile, strain J7-2 is capable of growth on synthetic media without amino acid supplements, and the biosynthetic pathways for 20 amino acids have been predicted in its genome, representing an ideal model for functionally dissecting amino acid biosynthesis pathways in haloarchaea (20). The strain J7-2 genome also contains three CRISPR loci (CRISPR1, CRISPR2, and CRISPR3), and the CRISPR1 locus is preceded by a full set of *cas* genes of the type I-B CRISPR-Cas system (20). Previously, a chemical mutagenesis of strain J7-2 generated arginine and lysine auxotrophs with mutations in *argC* and *dapD*, respectively, to develop a genetic manipulation system for this haloarchaeon (18). In this study, an endogenous CRISPR-Cas system-based genome editing method for strain J7-2 was established and used for dissection of the biosynthetic pathways of arginine and lysine, focusing on the functions of ArgW and ArgX in arginine biosynthetic pathway of haloarchaea. The possible evolutionary relationship between arginine and lysine biosynthetic pathways in haloarchaea is discussed.

## RESULTS

### Harnessing the endogenous CRISPR-Cas system for genome editing in strain J7-2.

**FIG 2:**
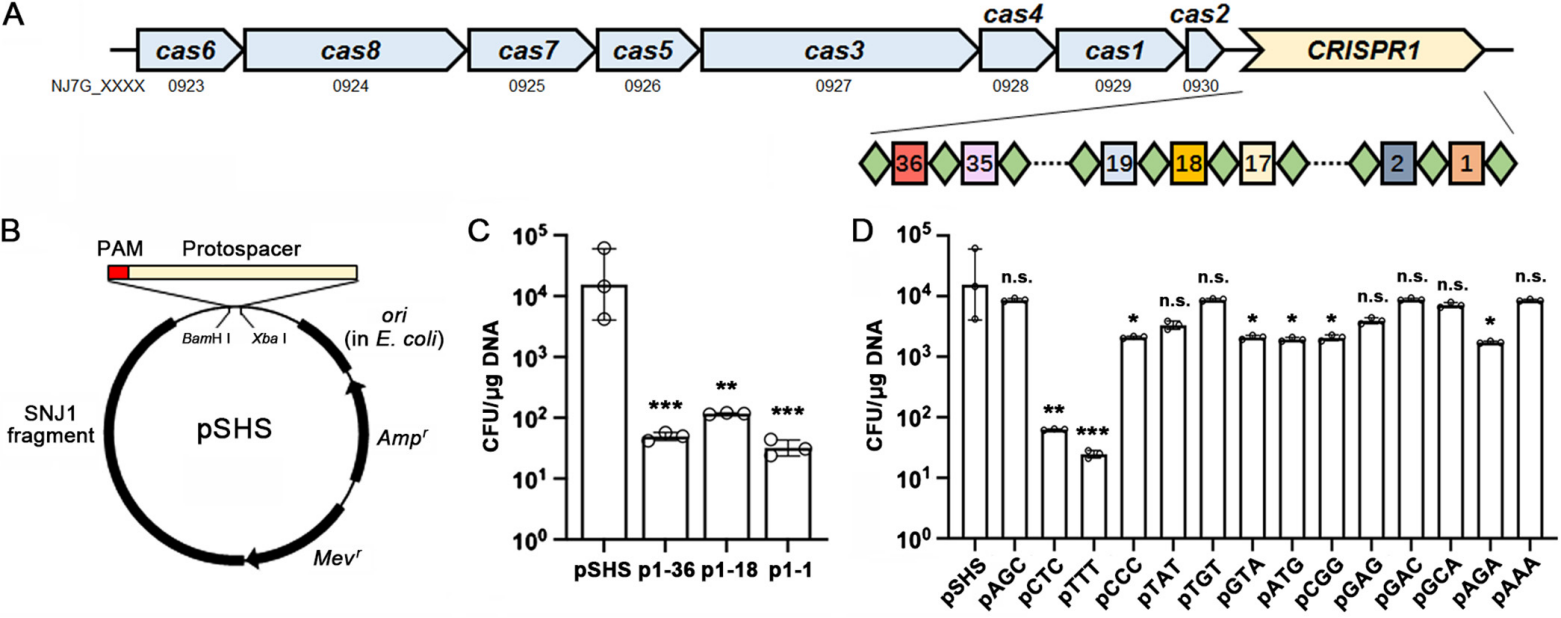
Challenging the CRISPR-Cas system of strain J7-2 with invader plasmids. (A) Schematic representation of the type I-B CRISPR-Cas system of strain J7-2. The Cas genes (*cas1* to *cas8*) and CRISPR1 are drawn to scale as arrows. The accession numbers of the Cas genes are shown below the arrows. Rectangles and diamonds represent the spacers and direct repeats, respectively. (B) Schematic representation of the invader plasmid. The invader DNA fragment comprising the PAM and protospacer was inserted into the vector pSHS to construct the invader plasmid for transformation of strain J7-2. (C) Transformation efficiencies of strain J7-2 by invader plasmids carrying the same PAM (TTC) and different protospacers matching spacer1-36, spacer1-18, and spacer1-1 of CRISPR1, respectively; (D) Transformation efficiencies of invader plasmids carrying the same protospacer (p1-36) and different trinucleotides as PAMs. The values are expressed as means ± standard deviation (SD) from three independent experiments (***, *P* < 0.001; **, *P* < 0.01; *, *P* < 0.05; n.s., no significance; calculated by Student's *t* test) (C and D), and the raw data are listed in Table S4.

The type I-B CRISPR-Cas system of strain J7-2 contains eight *cas* genes (*cas1* to *cas8*), and the CRISPR1 array is comprised of 37 repeats and 36 spacers serving as the main repository of guiding RNAs (Fig. 2A). To validate the function of the CRISPR-Cas system of strain J7-2, a plasmid challenge assay was performed by using invader plasmids carrying a spacer-matching artificial protospacer and a protospacer adjacent motif (PAM) (Fig. 2B). The trinucleotide TTC, a functional PAM verified in *Hfx. volcanii* (42) and *Har. hispanica* (43), was used as the PAM, and three spacers of the CRISPR1 array (spacer1-36, spacer1-18, and spacer1-1) of strain J7-2 were chosen as the protospacers to construct invader plasmids p1-36, p1-18, and p1-1, respectively, using the plasmid pYC-SHSmcs (41, 44) (here, abbreviated pSHS) as a vector (Fig. 2B). Subsequent transformation of strain J7-2 by the three invader plasmids resulted in an ~100-fold decrease in transformation rate compared with that by the parental plasmid pSHS (Fig. 2C). It was suggested that only those sequences that led to an at least 100-fold decrease in transformation rates in the plasmid challenge assay could be regarded as a functional PAM, because transformation rates cannot be determined very accurately (42, 45, 46). In this context, TTC is a functional PAM and the type I-B CRISPR-Cas system is active in strain J7-2.

Besides TTC, additional 14 trinucleotides were also tested for their potential as a functional PAM, with spacer1-36 being used as a protospacer in the constructed invader plasmids. The plasmid challenge assay showed that, among the 14 trinucleotides only CTC and TTT triggered a decrease in transformation rate by more than 100-fold (Fig. 2D) compared with pSHS without PAM and protospacer (Fig. 2C), indicating that CTC and TTT are also functional PAMs for strain J7-2.

The gene *crtB*, which encodes a phytoene synthase required for biosynthesis of carotenoids (e.g., β-carotene and bacterioruberin) in haloarchaea (47), has been used as the target gene to establish the endogenous CRISPR-Cas system-mediated gene knockout technique for *Har. hispanica*, and the Δ*crtB* deletion mutant (white) could be easily distinguished from the wild type (red) based on colony color phenotype (34). Accordingly, *crtB* (NJ7G_1607) was selected as the target gene to develop the endogenous CRISPR-Cas system-based gene editing technique in strain J7-2.

**FIG 3:**
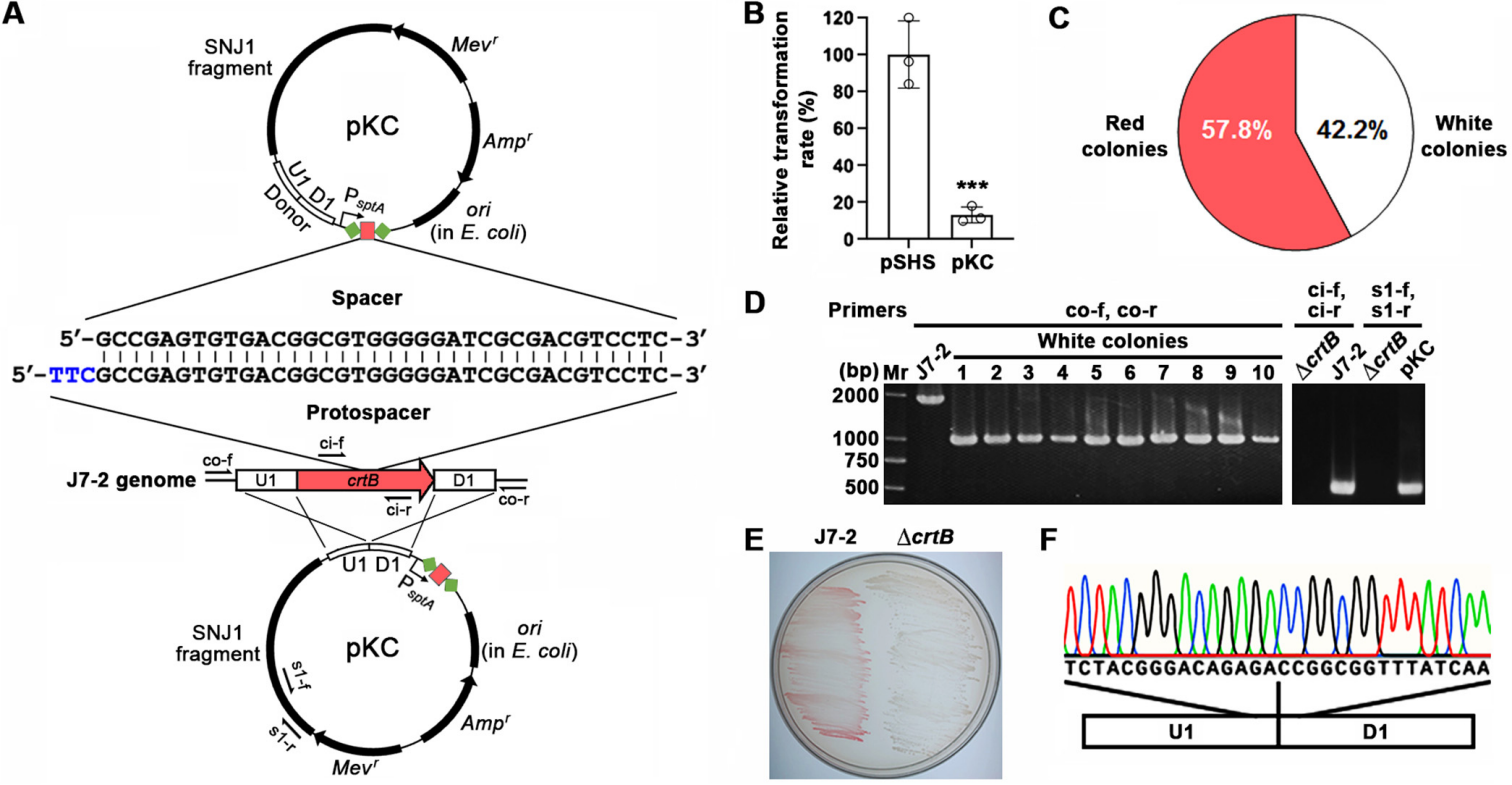
Knockout of *crtB* by endogenous CRISPR-Cas system in strain J7-2. (A) Schematic representation of pKC-mediated knockout of *crtB* in the strain J7-2 genome. The artificial mini-CRISPR on the plasmid pKC contains two repeats (green diamonds) separated by a spacer (red rectangle) matching a PAM (TTC [in blue])-preceded protospacer within *crtB* on strain J7-2 genome. The mini-CRISPR is driven by the *sptA* promoter (P*_sptA_*). The donor (U1 and D1) and the positions of the primers (co-f, co-r, ci-f, ci-r, s1-f, and si-r) used for PCR analysis are shown. (B) Comparison of the transformation rates of strain J7-2 by plasmids pSHS and pKC. The transformants were grown on 18% MGM plates containing 5 μg/mL mevinolin, and the values are expressed as means ± SD from three independent experiments (***, *P* < 0.001; calculated by Student's *t* test). The raw data are listed in Table S5. (C) Percentages of white and red colonies obtained when strain J7-2 was transformed by pKC. The values are calculated based on 462 pKC transformants. (D) PCR analyses of 10 randomly selected white colonies of pKC transformants and the Δ*crtB* mutant strain. The genomic DNAs of strain J7-2 and plasmid pKC were used as controls. (E) Color phenotypes of the J7-2 and Δ*crtB* strains grown on an 18% MGM plate; (F) Representative of the chromatograph of the DNA sequencing result of the Δ*crtB* strain.

An artificial mini-CRISPR, comprising two repeats separated by a spacer matching a PAM (TTC)-preceded sequence (protospacer) within *crtB*, as well as a donor DNA fragment were inserted into pSHS, yielding the self-targeting plasmid pKC; the mini-CRISPR is under the control of the *sptA* promoter (48) (Fig. 3A). Similar to the case of *Har. hispanica* (34), the transformation of strain J7-2 by pKC led to an ~10-fold decrease in transformation rate compared with that by pSHS (Fig. 3B), reflecting the cytotoxicity of the self-targeting CRISPR. Among 462 pKC transformants, 42.2% and 57.8% formed white and red colonies, respectively (Fig. 3C). By PCR analysis using the primers co-f and co-r (Fig. 3A), all of the 10 randomly selected white colonies showed the expected ~1-kb amplicon (Fig. 3D), suggesting that *crtB* has been deleted in these strains. Colony 4 (Fig. 3D) was chosen for curing of the self-targeting plasmid, and its subculture was subjected to PCR analysis using the primer pairs ci-f/ci-r and s1-f/s1-r, matching target sequences within *crtB* in the strain J7-2 genome and the plasmid pKC, respectively (Fig. 3A), showing that neither *crtB* nor pKC is present in this strain (Δ*crtB* mutant). The lawn color phenotype (Fig. 3E) and DNA sequencing result (Fig. 3F) also confirmed that *crtB* has been deleted from the genome in the Δ*crtB* mutant. These results indicate that the endogenous CRISPR-Cas system could be harnessed for genome editing in strain J7-2.

### Genes and enzymes involved in arginine and lysine biosynthesis in strain J7-2.

**FIG 4:**
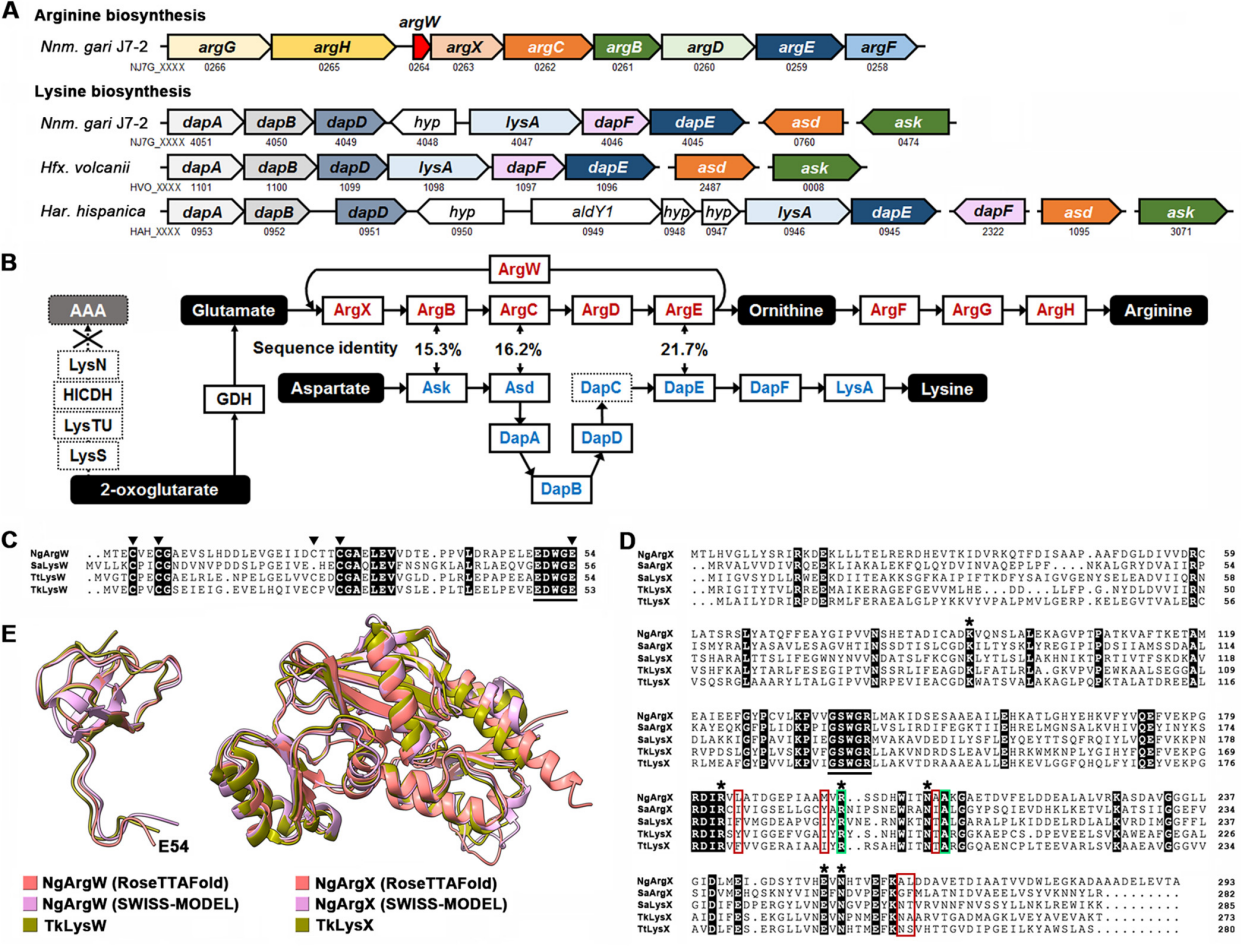
Genes and some enzymes involved in arginine and lysine biosynthesis of strain J7-2 and their homologs. (A) Predicted arginine and lysine biosynthetic gene clusters in strain J7-2 and some haloarchaea. Gene names and their locus tag numbers are shown, and “*hyp*” represents hypothetical genes. (B) Predicted arginine and lysine biosynthetic pathways in strain J7-2. The missing DapC in the lysine biosynthetic pathway and the missing enzymes for α-aminoadipate (AAA) biosynthesis are boxed by dotted lines. The amino acid sequence identity shared by ArgB and Ask, ArgC and Asd, as well as ArgE and DapE, respectively, are shown. (C) Amino acid sequence alignment of strain J7-2 ArgW (NgArgW [AFO55519]) with its homologs from S. acidocaldarius (SaLysW [ALU31683]), T. thermophilus (TtLysW [BCZ92879]), and T. kodakarensis (TkLysW [WP_011249234]). The conserved Zn^2+^-binding Cys residues and C-terminal Glu residue are indicated by arrowheads. The C-terminal EDWGE motif is underlined. (D) Amino acid sequence alignment of strain J7-2 ArgX (NgArgX [AFO55518]) with its homologs from S. acidocaldarius (SaArgX [WP_011278435] and SaLysX [WP_015385490]), T. kodakarensis (TkLysX [WP_011249233]), and T. thermophilus (TtLysX [WP_011173917]). The conserved residues of the active site are indicated by asterisks. The 5-amino-acid signature motif and two highly conserved residues involved in substrate recognition are boxed in red and green, respectively. The C-terminal GSWGR motif is underlined. (E) Superimpositions of structure models of NgArgW (left panel) and NgArgX (right panel) predicted by RoseTTAFold and SWISS-MODEL with the crystal structures of TkLysW (5K2M) and TkLysX (5K2M), respectively. The C-terminal Glu residue (E54) of NgArgW is indicated. The crystal structures of ArgW (3VPB) and ArgX (3VPB) of Sulfolobus tokodaii were used as the templates for homology modeling of NgArgW and NgArgX by SWISS-MODEL, respectively.

Strain J7-2 contains a full set of predicted genes involved in arginine biosynthesis (Fig. 4A); the products of *argW*, *argX*, *argB*, *argC*, *argD*, and *argE* are predicted to convert glutamate to ornithine, and those of *argF*, *argG*, and *argH* are predicted to convert ornithine to arginine (Fig. 4B). We noticed that ArgW, ArgX, ArgB, ArgC, ArgD, and ArgE of strain J7-2 share high sequence identities not only with those of the arginine biosynthetic pathway in other haloarchaea such as *Har. hispanica* (~63 to 78%) but also with the lysine biosynthesis enzymes of T. thermophilus (~36 to 54%); meanwhile, these enzymes are homologous to the bifunctional enzymes of S. acidocaldarius and T. kodakarensis involved in biosynthesis of both lysine and ornithine, with sequence identities of ~25 to 53% (Table 1). The LysW of T. thermophilus (TtLysW), S. acidocaldarius (SaLysW), or T. kodakarensis (TkLysW) possesses four or three Zn^2+^-binding Cys residues and a EDWGE motif containing the C-terminal Glu residue to covalently link the amino group of AAA or glutamate (6, 11, 12, 49), and these crucial residues are conserved in the *Nnm. gari* J7-2 ArgW (NgArgW) (Fig. 4C). The six residues of the active site in LysX and ArgX of T. thermophilus (TtLysX), S. acidocaldarius (SaLysX and SaArgX), and T. kodakarensis (TkLysX) are conserved in the strain J7-2 ArgX (NgArgX) (Fig. 4D). NgArgX also possesses a GSWGR motif that recognizes the EDWGE motif of LysW/ArgW proteins (11, 12) (Fig. 4C and D). Additionally, the predicted overall structures of NgArgW and NgArgX, generated by either RoseTTAFold (50) or SWISS-MODEL (51), are well superimposed with the crystal structures of their homologs (TkLysW and TkLysX) of T. kodakarensis (12) (Fig. 4E). These pieces of evidence raise the possibility that the predicted arginine biosynthetic pathway of strain J7-2 may be a bifunctional route for biosynthesis of not only arginine but also lysine via the AAA pathway, but the strain J7-2 genome lacks the genes encoding the enzymes (LysS, LysTU, homoisocitrate dehydrogenase, and LysN) required for *de novo* biosynthesis of AAA from 2-oxoglutarate (Fig. 4B). Additionally, in LysX/ArgX proteins a 5-amino-acid signature motif and two highly conserved residues (Arg and Ala) are involved in substrate recognition (12), while the 5-amino-acid signature motif of NgArgX is different from those of SaArgX, SaLysX, TtLysX, and TkLysX (Fig. 4D) but is highly conserved in haloarchaeal ArgX proteins (see Fig. S1A in the supplemental material).

**TABLE 1:** Amino acid sequence identities shared by the arginine biosynthetic enzymes of strain J7-2 and their homologs

Source	Pathway	% of identity to^*a*^:								
		ArgW (LysW)	ArgX (LysX)	ArgB (LysZ)	ArgC (LysY)	ArgD (LysJ)	ArgE (LysK)	ArgF	ArgG	ArgH
*Har. hispanica*	Arginine	78.2	71.6	64.4	76.9	63.1	64.3	63.4	69.5	64.2
T. thermophilus	Lysine	53.6	41.2	38.7	45.0	43.1	35.5			
S. acidocaldarius	Arginine/lysine	39.7	28.3/28.2	28.3	41.8	35.7	25.4	38.0	34.8	28.7
T. kodakarensis	Ornithine/lysine	52.7	35.3	28.9	44.2	37.0	31.8	50.9		

a The names of the enzymes involved in lysine biosynthesis are listed in parentheses.

By using the crystal structure of the LysW-ADP-glutamate-bound complex of ArgX from Sulfolobus tokodaii (StArgX) (11) as the template, the homology modeling of NgArgX shows that four of the six hydrogen bonds formed between glutamate and the substrate recognition residues of StArgX are conserved in the glutamate-binding model of NgArgX (Fig. S1B). It seems that the substrate recognition mechanism of NgArgX is similar to that of StArgX, although the 5-amino-acid signature motifs of the two enzymes are different.

In strain J7-2, a gene cluster (*dapA*, *dapB*, *dapD*, *hyp4048*, *lysA*, *dapF*, and *dapE*) and two genes (*ask* and *asd*) at other loci are predicted to constitute the DAP pathway for lysine biosynthesis (Fig. 4A), although the gene encoding DapC is missing (Fig. 4B). These genes, except *dapC* and *hyp4048*, are conserved in other haloarchaea such as *Hfx. volcanii* and *Har. hispanica* (Fig. 4A). It is known that the products of four genes (*ask*, *asd*, *dapC*, and *dapE*) of the DAP pathway are evolutionarily related to the arginine biosynthetic enzymes encoded by *argB*, *argC*, *argD*, and *argE*, respectively (1, 3). In strain J7-2, however, Ask, Asd, and DapE share only low sequence identities (15.3 to 21.7%) with their counterparts (ArgB, ArgC, and ArgE) in the arginine biosynthetic pathway (Fig. 4B), reflecting a high evolutionary divergence between the two sets of enzymes.

### *argW* and *argX* are essential for biosynthesis of arginine rather than lysine in strain J7-2.

**FIG 5:**
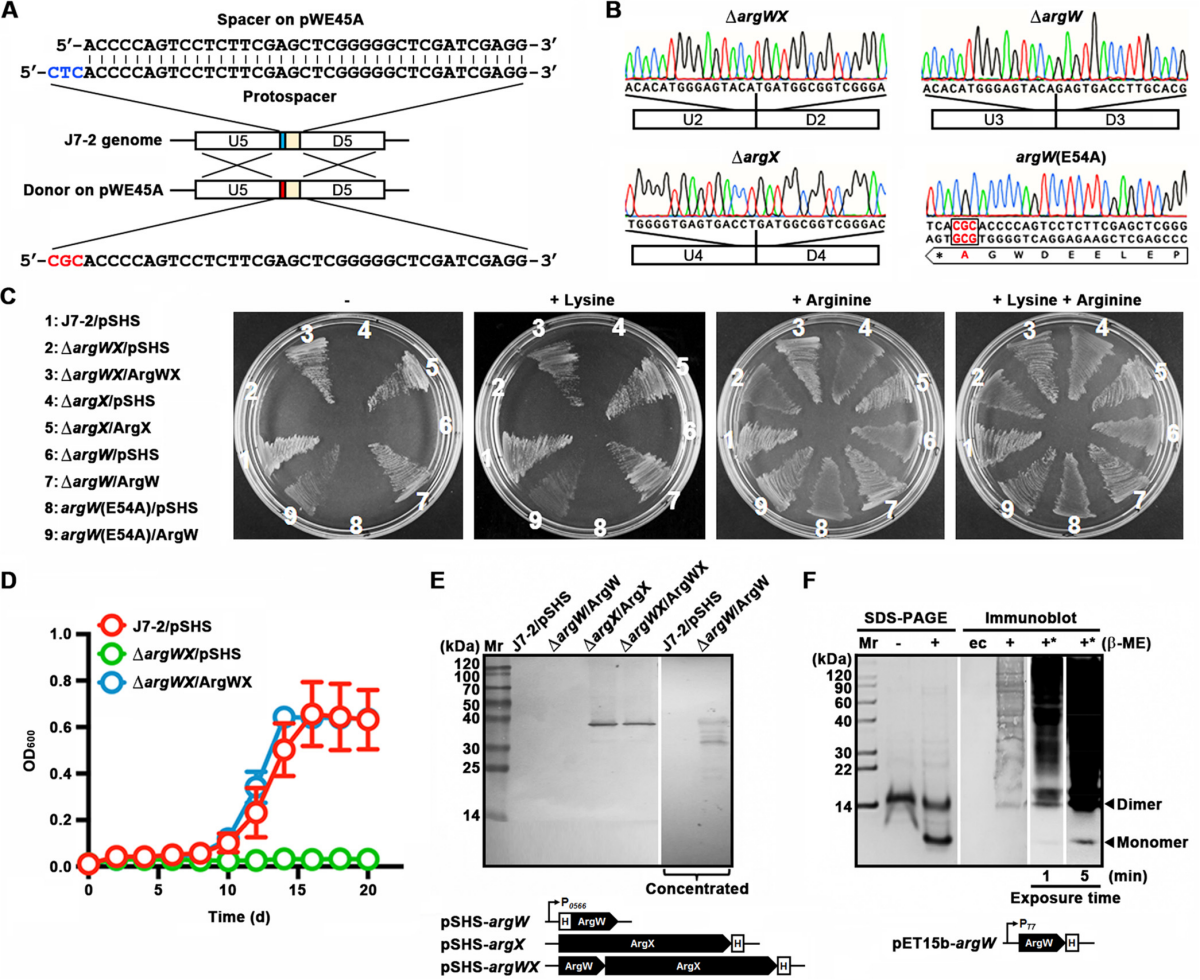
Roles of NgArgW and NgArgX in arginine biosynthesis in strain J7-2. (A) Schematic representation of the *in situ* mutation of *argW* in strain J7-2. The self-targeting plasmid pWE45A carries a spacer matching a PAM (CTC [in blue])-preceded protospacer corresponding to the 3′-end region of *argW* on strain J7-2 genome, and the donor DNA (U5 and D5) in pWE45A contains a mutation (CGC [in red]) leading to the replacement of the Glu codon (GAG) by the Ala codon (GCG). (B) Representatives of chromatographs of DNA sequencing results of target mutant strains. (C) Amino acid requirements of strain J7-2 and its derivatives. Cells of each strain were streaked on SM plates without (−) or with (+) 0.1 mM lysine and/or arginine and grown at 37°C. Photographs were taken after 6 days. (D) Growth curves of target strains. Each strain was cultivated in liquid SM at 37°C. The values are expressed as means ± SD from three independent experiments. (E) Detection of recombinant proteins in complementary strains. The cell extracts of the indicated strains grown in liquid SM were subjected to anti-His tag immunoblot analysis after SDS-PAGE using a Tris-tricine buffer system. The cell extracts of strain J7-2 and the Δ*argW*/ArgW complementary strain were also subjected to Ni-NTA affinity chromatography, and the elution fractions were used as concentrated samples for immunoblot analysis. (F) SDS-PAGE and immunoblot analyses of purified recombinant NgArgW produced in E. coli. Purified NgArgW was subjected to urea-SDS-PAGE using a Tris-tricine buffer system in the absence (−) or presence (+) of 1% β-ME, followed by anti-His tag immunoblot analysis, with the cell extract of E. coli carrying a blank vector as the control (ec). Blotted proteins were also visualized and photographed by using ECL (lanes indicated by asterisks) with an exposure time of 1 or 5 min. Schematic representation of promoter (*P*_0566_ or *P*_T7_)-preceded recombinant genes with His tag (H) coding sequence on expression plasmids are shown (E and F, bottom).

The endogenous CRISPR-Cas system-based genome editing method was employed to delete *argW* and *argX* of strain J7-2 simultaneously or separately, generating the Δ*argWX*, Δ*argX*, and Δ*argW* mutant strains. To probe the role of the C-terminal residue Glu^54^ of NgArgW, we constructed the plasmid pWE54A, carrying a spacer matching a CTC-preceded protospacer at the 3′ end of *argW* and a donor DNA with a codon mutation leading to the replacement of Glu^54^ by Ala (E54A), and used it to construct the *argW*(E54A) mutant strain (Fig. 5A). All of the four mutant strains were verified by DNA sequencing (Fig. 5B). In contrast to strain J7-2, the four mutants were unable to grow on the synthetic medium (SM) plate without amino acid supplements or supplemented only with lysine, but grew when supplemented with arginine (Fig. 5C). By using pSHS as the vector, we constructed the complementary plasmid expressing either or both NgArgW and NgArgX driven by the promoter (P*_0566_*) of a high-abundance blue copper domain protein (NJ7G_0566) of strain J7-2 (52) (Fig. 5E, bottom). Similar to strain J7-2, the Δ*argWX*/ArgWX, Δ*argX*/ArgX, Δ*argW*/ArgW, and *argW*(E54A)/ArgW complementary strains were able to grow on SM plates either with or without arginine or lysine (Fig. 5C). In liquid SM, the Δ*argWX*/ArgWX complementary strain rather than the Δ*argWX* mutant harboring the blank vector pSHS could grow and show a similar growth profile to strain J7-2 carrying pSHS under the same cultivation condition (Fig. 5D). These results suggest that *argW* and *argX* are indispensable for biosynthesis of arginine rather than lysine and that the C-terminal residue Glu^54^ of NgArgW is crucial for arginine biosynthesis in strain J7-2.

The results of anti-His tag immunoblot analyses showed that recombinant NgArgX with a C-terminal His tag could be detected in the cell extracts of Δ*argWX*/ArgWX and Δ*argX*/ArgX strains grown in liquid SM; however, NgArgW was not detected in the extract of the Δ*argW*/ArgW strain (Fig. 5E), probably due to a low expression level of this protein. Subsequently, the cell extract of the Δ*argW*/ArgW strain was subjected to nickel-nitrilotriacetic acid (Ni-NTA) affinity chromatography to concentrate the recombinant NgArgW with an N-terminal His tag for immunoblot analysis, revealing multiple bands with apparent molecular weights (AMWs [~25 to 40 kDa]) much higher than predicted molecular weight of recombinant NgArgW (6.7 kDa) (Fig. 5E). Considering that NgArgW contains four Cys residues, we reasoned that the observed higher-AMW forms of NgArgW may have resulted from the formation of intermolecular disulfide bonds during the electrophoresis. To test this possibility, recombinant NgArgW with a C-terminal His tag was produced in E. coli and purified by Ni-NTA affinity chromatography, followed by SDS-PAGE analysis in the presence of 8 M urea. Before treatment with β-mercaptoethanol (β-ME), purified NgArgW showed an AMW of ~14 kDa; after β-ME treatment, a portion of the 14-kDa NgArgW molecules were converted into monomeric forms (~7 kDa) (Fig. 5F). Besides the 14-kDa NgArgW, higher-AMW forms of NgArgW were also detected by immunoblot analysis (Fig. 5F), which suggests the formation of intermolecular disulfide bonds between two or more NgArgW molecules during the electrophoresis, even when the protein sample was pretreated with β-ME. In contrast to the 14-kDa NgArgW, the 7-kDa NgArgW monomer was not revealed by anti-His tag immunoblot analysis (Fig. 5F). When the blotted proteins were visualized by using enhanced chemiluminescence (ECL), the 7-kDa NgArgW monomer could be detected (Fig. 5F). These results confirm that recombinant NgArgW and NgArgX have been expressed to fulfil their functions in the complementary strains.

### The arginine and lysine biosynthetic pathways are independent of each other in strain J7-2.

**FIG 6:**
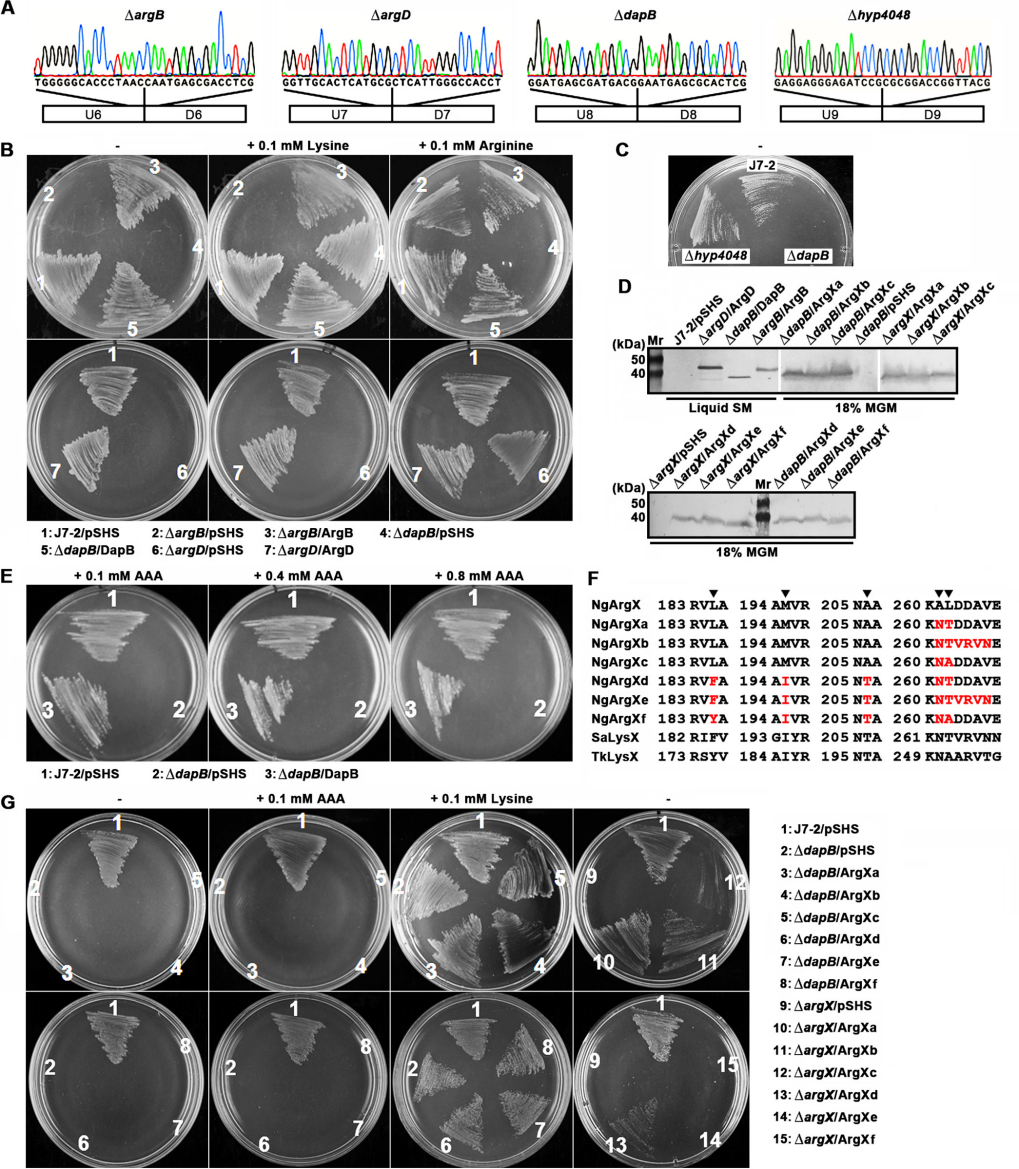
Functional analyses of selected genes in arginine and lysine biosynthetic pathways of strain J7-2. (A) Representative chromatographs of DNA sequencing results of target mutant strains; (B, C, E, and G) amino acid requirements of strain J7-2 and its derivatives. Cells of each strain were streaked on SM plates without (−) or with (+) lysine, arginine, or AAA and grown at 37°C. Photographs were taken after 6 days. (D) Detection of recombinant proteins in complementary strains. The cell extracts of the indicated strains grown in liquid SM or 18% MGM were subjected to anti-His tag immunoblot analysis after SDS-PAGE using a Tris-glycine buffer system. (F) Alignment of amino acid sequences around the 5-amino-acid signature motifs of NgArgX and its variants, SaLysX, and TkLysX. The signature motif is indicated by arrowheads. The mutated residues in the variants of NgArgX are marked in red.

To investigate whether the predicted DAP pathway is functional in strain J7-2 and to probe possible interconnection between the lysine and arginine biosynthetic pathways, four genes of the two pathways were chosen to construct the Δ*dapB*, Δ*hyp4048*, Δ*argB*, and Δ*argD* mutants via the endogenous CRISPR-Cas system-based genome editing method, and the resulting mutants were confirmed by DNA sequencing (Fig. 6A).

The Δ*dapB* mutant was unable to grow on the SM plate without amino acid supplements but grew well with lysine supplement; in contrast, the Δ*dapB*/DapB complementary strain was able to grow on the SM plate in either the absence or presence of lysine (Fig. 6B). Meanwhile, recombinant DapB could be detected in Δ*dapB*/DapB by immunoblot analysis (Fig. 6D). In the DAP pathway gene cluster of strain J7-2, the gene *hyp4048* encodes a hypothetical protein (NJ7G_4048) (Fig. 4A). However, the Δ*hyp4048* mutant grew as well as strain J7-2 on the SM plate without amino acid supplements (Fig. 6C). These results suggest that *dapB* but not *hyp4048* is necessary for lysine biosynthesis via the DAP pathway in strain J7-2.

Despite their evolutionary relationships, the DAP pathway enzymes Ask, Asd, and DapE of strain J7-2 share only 15.3 to 21.7% sequence identities with their counterparts (ArgB, ArgC, and ArgE) in the arginine biosynthetic pathway (Fig. 4B), implying that the two sets of enzymes have become highly specialized for corresponding pathways during evolution. In support of this, the Δ*argB* mutant strain was unable to grow on the SM plate without arginine supplement; in contrast, the Δ*argB*/ArgB complementary strain grew well under the same condition (Fig. 6B), and recombinant ArgB was detected in the Δ*argB*/ArgB strain by immunoblot analysis (Fig. 6D). These results suggest that the loss of *argB* in Δ*argB* cannot be compensated for by its counterpart *ask* in the DAP pathway of strain J7-2.

Because *dapC* is missing in haloarchaea, it is assumed that haloarchaeal ArgD is synonymous with DapC and may act as not only an *N*-acetylornithine aminotransferase for arginine biosynthesis but also an *N*-succinyl-l, l-diaminopimelate aminotransferase for lysine biosynthesis (19). To test this possibility, the Δ*argD* mutant was analyzed for its amino acid requirements for growth. On SM plates, the Δ*argD* mutant could grow only in the presence of arginine but did not grow without amino acid supplements or with lysine supplement, while the Δ*argD*/ArgD complementary strain did not require amino acid supplements for growth (Fig. 6C). Immunoblot analysis also revealed the recombinant ArgD in the Δ*argD*/ArgD strain (Fig. 6D). These results indicate that ArgD participates only in arginine biosynthesis and that the function of the missing DapC is accomplished by a yet unknown aminotransferase in strain J7-2.

Although the strain J7-2 genome lacks the genes encoding the enzymes for *de novo* biosynthesis of AAA from 2-oxoglutarate (Fig. 4B), there is a possibility that strain J7-2 may utilize external AAA to synthesize lysine via the arginine biosynthetic pathway, of which the enzymes share high sequence identities with their counterparts of the LysW-mediated AAA pathways in T. thermophilus, S. acidocaldarius, and T. kodakarensis (Table 1). To test this possibility, the Δ*dapB* mutant strain, in which the DAP pathway has been blocked, was inoculated on SM plates supplemented with different concentrations of AAA, but no growth of this mutant was observed, whereas the Δ*dapB*/DapB complementary strain grew well under same conditions (Fig. 6E). Therefore, the arginine biosynthetic pathway of strain J7-2 cannot act as a bifunctional route for biosynthesis of both arginine and lysine, and lysine is synthesized only via the DAP pathway.

### NgArgX is a key determinant of substrate specificity of the ArgW-mediated arginine biosynthetic pathway.

It was reported that the 5-residue signature motif defines substrate specificity of LysX/ArgX proteins for glutamate and AAA, respectively (11, 12) (Fig. 4D). In order to investigate whether the substrate specificity of NgArgX could be altered and used for lysine biosynthesis from AAA, we constructed four variants (NgArgXa, NgArgXc, NgArgXd, and NgArgXf) of NgArgX by replacing some or all of the residues in the signature motif with the corresponding residues of monofunctional SaLysX or bifunctional TkLysX; an additional four residues adjacent to the signature motif were also replaced by corresponding residues of SaLysX, yielding the variants NgArgXb and NgArgXe (Fig. 6F). However, the Δ*dapB* strains carrying the expression plasmids for the six mutated NgArgX proteins, respectively, could grow on SM plates supplemented with lysine but not on that supplemented with AAA (Fig. 6G), although the six variants could be produced in the Δ*dapB* mutant grown in modified growth medium with 18% total salts (18% MGM) by immunoblot analysis (Fig. 6D). Moreover, all of the six variants could be produced in the Δ*argX* mutant grown in 18% MGM (Fig. 6D), while four of them (NgArgXa, NgArgXb, NgArgXc, and NgArgXd) could rescue the growth defect of the Δ*argX* mutant on SM plates without amino acid supplements (Fig. 6G), indicating that the substrate specificity of the four variants has not been altered by modification of the signature motif. Notably, the variants NgArgXe and NgArgXf could not rescue the growth defect of the Δ*argX* mutant on SM plates without amino acid supplements (Fig. 6G), suggesting that the mutations in the signature motif of the two variants not only are unable to endow them with the ability to act on AAA but also lead to the loss of their specificity for glutamate.

**FIG 7:**
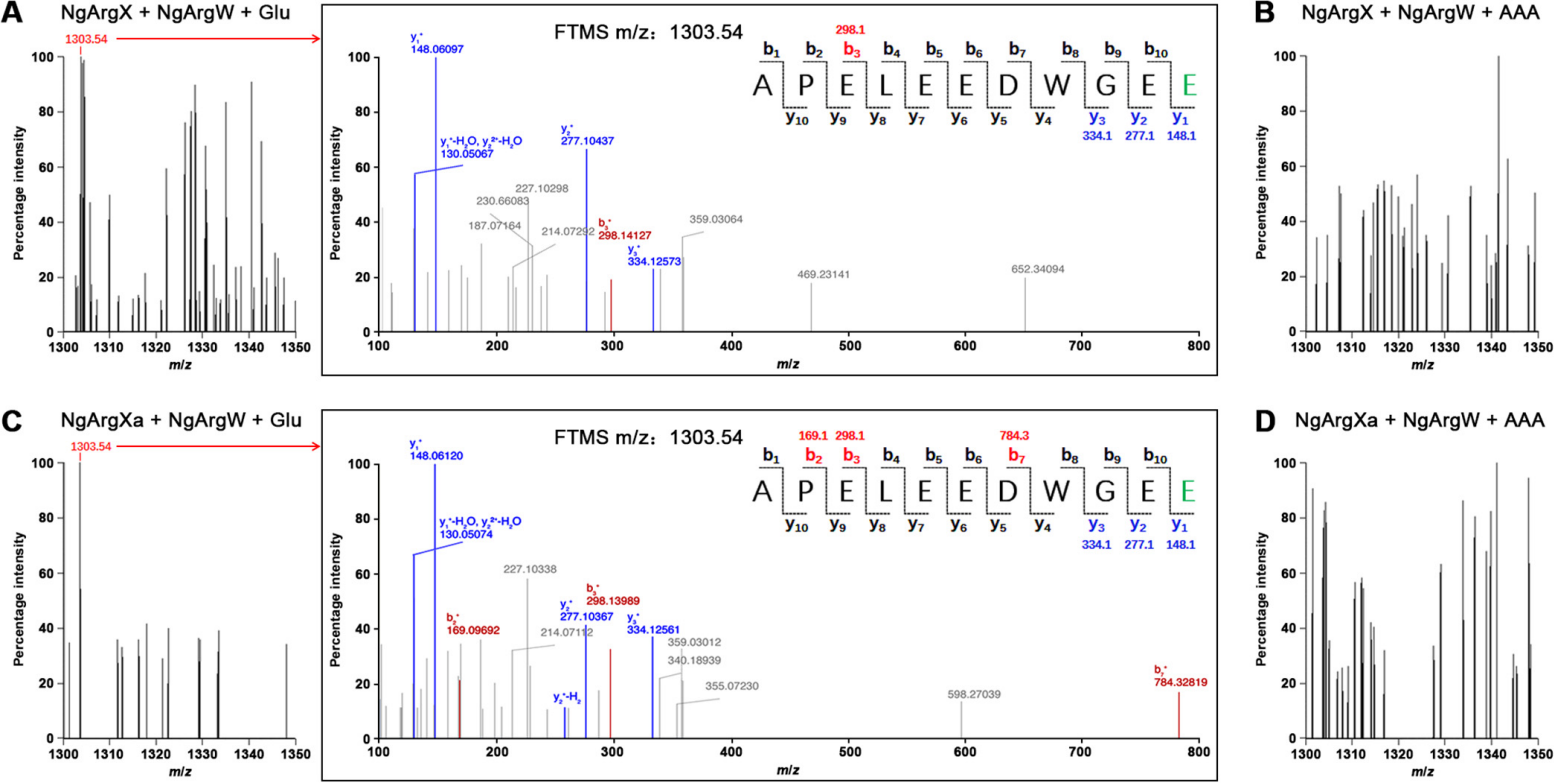
LC-MS/MS analysis of trypsin-digested NgArgW derivatives. (A to D) Mass spectra of trypsin-digested NgArgW derivatives in different reaction mixtures as indicated. The MS/MS spectrum of the ion at *m/z* = 1,303.54 in panel A or C is boxed to the right, and the glutamate covalently linked to the C-terminal residue Glu^54^ of NgArgW is marked in green.

We next investigated whether NgArgX and its variant NgArgXa can catalyze the covalent linkage of glutamate and/or AAA with NgArgW *in vitro*. Recombinant NgArgW with an N-terminal His tag was purified from E. coli, while NgArgX and NgArgXa were expressed and partially purified from the Δ*argWX* mutant (Fig. S2). NgArgX or NgArgXa was mixed with NgArgW and the substrate glutamate or AAA, and after the reaction the mixture was treated with trypsin, followed by liquid chromatography-tandem mass spectrometry (LC-MS/MS) analysis. When glutamate was used as the substrate, a peptide corresponding to the NgArgW-derived C-terminal peptide ^45^APELEEDWGE^54^ plus Glu (1,303.54 Da) was identified in the reaction mixture containing either NgArgX or NgArgXa, and the covalent linkage of the substrate glutamate with the C-terminal residue Glu^54^ of NgArgW was verified by LC-MS/MS analysis (Fig. 7A and C). In contrast, when AAA was used as the substrate the peptide ^45^APELEEDWGE^54^ plus AAA (theoretical mass of 1,317.63 Da) was not identified in the reaction mixture containing either NgArgX or NgArgXa (Fig. 7B and D). These results suggest that both NgArgX and NgArgXa specifically act on glutamate rather than AAA.

## DISCUSSION

### Endogenous CRISPR-Cas system-based genome editing in *Nnm. gari* J7-2.

In this study, the CRISPR-Cas system of *Nnm. gari* J7-2 has been verified to be functional and successfully harnessed for genome editing in this haloarchaeon. Because CRISPR-Cas systems are widely distributed in prokaryotes, endogenous CRISPR-Cas system-based genome editing is emerging as a new strategy for editing bacterial and archaeal genomes, but very few reports are available on successfully exploiting haloarchaeal CRISPR-Cas systems for genome editing. For instance, *Hfx*. *volcanii* is highly tolerant to self-targeting of the genome by its native CRISPR-Cas system, and exploiting endogenous CRISPR-Cas system-based genome editing in *Hfx*. *volcanii* is impeded, most likely due to an accurate genome repair via homologous recombination in this polyploid haloarchaeon (34). Nevertheless, endogenous CRISPR-Cas system-mediated gene repression can be achieved in *Hfx*. *volcanii* after deleting the genes encoding the nuclease Cas3 for target cleavage and the RNase Cas6 for pre-CRISPR RNA (pre-crRNA) processing (53). In contrast to the case of *Hfx. volcanii*, CRISPR-mediated self-targeting is highly cytotoxic in the polyploid haloarchaeon *Har. hispanica*, and endogenous CRISPR-Cas system-based genome editing could be readily achieved with a high efficiency (35). The discrepancy in tolerance to CRISPR-mediated self-targeting between *Hfx. volcanii* and *Har. hispanica* may derive from a possible difference in their chromosome copy numbers, DNA repair systems, and/or CRISPR-Cas systems (34, 35). Differing from *Hfx. volcanii* but similar to *Har. hispanica*, *Nnm. gari* J7-2 is sensitive to CRISPR-mediated self-targeting, facilitating the establishment of endogenous CRISPR-Cas system-based genome editing in this haloarchaeon.

It was observed that when deleting *crtB* by the endogenous CRISPR-Cas system-based genome editing method, only 42.2% of strain J7-2 transformants formed white colonies, while nearly all the colonies of *Har. hispanica* transformants showed a white phenotype (35), implying that the gene-editing efficiency in strain J7-2 is lower than that in *Har. hispanica*. Besides a possible difference in the activities of CRISPR-Cas systems between the two haloarchaea, the copy number of the self-targeting plasmid may be important for the gene-editing efficiency. In this study, the plasmid pSHS, which is present at about 1 copy per chromosome in *Nnm. gari* cells (44), was employed to construct the self-targeting plasmid pKC containing the mini-CRISPR and the donor DNA. Due to the relatively low copy number of pKC, the amount of the donor DNA and the expression level of the self-targeting crRNA may not be high enough for highly efficient knockout of *crtB* in the chromosome. Nevertheless, the use of a low-copy self-targeting plasmid for gene editing has the advantage of an efficient plasmid curing, as evidenced by the finding that the Δ*crtB* mutant lacking the self-targeting plasmid could be obtained after only two rounds of subculturing. In *Har. hispanica*, the endogenous CRISPR-Cas system-based genome editing system has been optimized in terms of the selection of PAM and the promoter of the mini-CRISPR, as well as the size of the donor DNA (35). Additionally, it was reported that the length of the spacer in the mini-CRISPR is related to the gene-editing efficiency of the endogenous CRISPR-Cas system in Clostridium tyrobutyricum (30). Future optimization of the corresponding elements of the self-targeting plasmid is expected to improve the endogenous CRISPR-Cas system-based genome editing efficiency in strain J7-2.

### Arginine and lysine biosynthesis in *Nnm. gari* J7-2.

By using the endogenous CRISPR-Cas system-based genome editing system, several genes predicted to be involved in the biosynthesis of arginine and lysine in strain J7-2 were genetically dissected. In contrast to the wild-type strain J7-2, the Δ*argW*, Δ*argX*, Δ*argB*, and Δ*argD* mutant strains display an arginine auxotrophic phenotype. Meanwhile, the mutagenesis of *argC* or *argH* in *Hfx. volcanii* or *Nnm. gari* J7-2 also generates an arginine auxotroph (18, 21, 22). These data confirm that the ArgW-mediated arginine biosynthetic pathway is functional and indispensable for arginine biosynthesis in haloarchaea. In bacteria, *N*-acetylglutamate synthase (ArgA) or ornithine acetyltransferase (ArgJ) is responsible for the acetylation of the α-amino group of glutamate to prevent intramolecular cyclization of intermediates during arginine biosynthesis (2). In hyperthermophilic archaea such as S. acidocaldarius and T. kodakarensis, the α-amino group of glutamate is covalently linked to the γ-carboxyl group of the C-terminal Glu residue of the carrier protein SaLysW or TkLysW to avoid intramolecular cyclization (11, 12). Similar to hyperthermophilic archaea, the haloarchaeon *Nnm. gari* J7-2 employs the carrier protein NgArgW to modify the α-amino group of glutamate. In support of this, the *argW*(E54A) mutant strain, in which the C-terminal residue Glu^54^ of NgArgW is replaced by alanine, shows an arginine auxotrophic phenotype. It appears that the modification of the α-amino group of glutamate with a carrier protein rather than an acetyl group is a common strategy employed by archaea for arginine biosynthesis.

It was noticed that the recombinant NgArgW, which has four Cys residues conserved in LysW-like proteins, tends to form intermolecular disulfide bonds by SDS-PAGE analysis. However, because the cytoplasm is normally maintained in a reducing state due to the presence of antioxidants such as thioredoxins, glutaredoxins, and glutathione and its analogs (54, 55), disulfide bond formation between NgArgW molecules is unlikely to occur in the cytoplasm. Moreover, in the crystal structure of TtLysW or TkLysW, a zinc atom is coordinated with the four Cys residues (12, 49). Accordingly, the four Cys residues in NgArgW most likely participate in Zn^2+^ binding rather than form disulfide bonds *in vivo*. In contrast, during the SDS-PAGE analysis, the structure of NgArgW is disrupted, allowing the exposure of Cys residues to form disulfide bonds under oxidative conditions.

Although resembling S. acidocaldarius and T. kodakarensis in employing the LysW/ArgW-mediated pathway for arginine biosynthesis, strain J7-2 differs from the two hyperthermophilic archaea in that it synthesizes lysine via the DAP pathway rather than the AAA pathway, as evidenced by the finding that the Δ*dapB* mutant strain shows a lysine auxotrophic phenotype. Similar to the case of strain J7-2, the isotopic labeling study on *Har. hispanica* (16) and the mutagenesis of *lysA* in *Hfx. volcanii* (21) show that the two haloarchaea utilize the DAP pathway for lysine biosynthesis. While *Har. hispanica* synthesizes lysine via the dehydrogenase subpathway (16), *Nnm. gari* J7-2 employs the succinylase subpathway for lysine biosynthesis since the mutation of *dapD* specific for this subpathway leads to lysine auxotrophy in this haloarchaeon (18). Although the genes encoding DapC and diaminopimelate dehydrogenase (Ddh), specific for the succinylase and dehydrogenase subpathways, respectively, have not been identified yet, it is evident that the DAP pathway is commonly employed by haloarchaea for lysine biosynthesis.

In Gram-negative bacteria, the DAP pathway not only is responsible for lysine biosynthesis but also plays a central role in cell wall synthesis since DAP is an essential precursor for peptidoglycan biosynthesis (3). For those bacteria lacking the DAP pathway, such as T. thermophilus and Deinococcus radiodurans (56), their cell wall does not have DAP but contains ornithine, an intermediate of the arginine biosynthetic pathway (57, 58). In the cases of S. acidocaldarius and T. kodakarensis, they also lack the DAP pathway but employ the LysW-mediated AAA pathway for lysine biosynthesis, and they have only a single S-layer as their cell wall (59, 60). It has been proposed that the maintenance of the DAP pathway for lysine biosynthesis, at least in bacteria, might be correlated with the appearance of the extant structure of their cell wall (3). However, this proposal is not applicable to haloarchaea, which have the S-layer as the only component of their cell wall (61) but retain the DAP pathway for lysine biosynthesis. It is presently unclear whether the haloarchaeal DAP pathway has any function other than lysine biosynthesis. The possibility that the intermediates of the DAP pathway may be involved in some cellular processes in haloarchaea cannot be excluded.

### The relationship between the arginine and lysine biosynthetic pathways in *Nnm. gari* J7-2.

Despite the evolutionary relationship between the arginine and lysine biosynthetic pathways, our results suggest that the biosynthetic pathway for arginine and the DAP pathway for lysine are independent of each other in strain J7-2. First, the Δ*argW*, Δ*argX*, Δ*argB*, and Δ*argD* mutant strains show only an arginine auxotrophic phenotype, while the Δ*dapB* mutant strain shows only a lysine auxotrophic phenotype. Second, the loss of the gene *argB*, which is necessary for arginine biosynthesis, cannot be compensated for by its counterpart *ask* in the DAP pathway. Third, the enzyme ArgD, which is assumed to be synonymous with DapC of the DAP pathway in haloarchaea (19), is not involved in lysine biosynthesis in strain J7-2. Finally, the strain J7-2 genome lacks the genes for *de novo* biosynthesis of AAA, and the DAP pathway-blocked Δ*dapB* strain is unable to grow in the presence of AAA; the replacement of the 5-amino-acid signature motif responsible for substrate specificity of NgArgX by that of SaLysX or TkLysX cannot endow the Δ*dapB* mutant with the ability to biosynthesize lysine from AAA. In *Hfx. volcanii*, the mutagenesis of the genes involved in arginine (*argB*, *argC*, *argD*, and *argH*) and lysine (*lysA*) biosynthesis also generates arginine and lysine auxotrophs, respectively (21, 22). In this context, the two sets of enzymes of the arginine and lysine biosynthetic pathways in haloarchaea have been highly specialized during evolution.

As in other haloarchaea, the gene *dapC* in the DAP pathway has not been identified in the strain J7-2 genome. Our results show that the ArgD of strain J7-2 participates only in biosynthesis of arginine rather than lysine. Similarly, the mutagenesis of *argD* in *Hfx. volcanii* generates an arginine auxotroph (21). These data suggest that haloarchaeal ArgD is a monofunctional enzyme specific for the arginine biosynthetic pathway and exclude the possibility that this enzyme is synonymous with DapC in haloarchaea (19). This is in contrast to the case of ArgD from E. coli, which acts as a bifunctional enzyme to catalyze the transamination reactions in both the arginine biosynthetic pathway and the DAP pathway (14). Given the fact that the succinylase subpathway is functional in strain J7-2, there should be a yet unknown aminotransferase gene to fulfil the function of the missing *dapC*; however, this unknown gene is likely to share a very low sequence identity with known *dapC* or may be a nonorthologous gene and remains to be identified in the future.

### Determinant role of NgArgX in substrate specificity of the ArgW-mediated arginine biosynthetic pathway.

Strain J7-2 utilizes the ArgW-mediated pathway to synthesize arginine but not lysine, although the enzymes of this pathway are homologs of those of the bifunctional LysW-mediated pathways for both lysine and arginine (or ornithine) in S. acidocaldarius and T. kodakarensis (11, 12). Considering that SaLysW could act as a common carrier protein of glutamate and AAA to mediate the biosynthesis of both arginine and lysine in S. acidocaldarius (11), the carrier protein NgArgW is unlikely to be crucial for determination of the preference of the ArgW-mediated pathway for glutamate rather than AAA as the substrate in strain J7-2. A more likely scenario is that the catalytic enzyme or enzymes in the ArgW-mediated pathway of strain J7-2 have become highly specific for glutamate and its derivatives to biosynthesize arginine, wherein the first enzyme of the pathway, NgArgX, plays a key role in determining the pathway specificity. For LysX/ArgX proteins from hyperthermophilic archaea, the replacement of two or four residues in the signature motif of bifunctional TkLysX by monofunctional LysX- or ArgX-type residues increases substrate preference of the variant for AAA or glutamate, respectively (12): the replacement of two residues in the signature motif of SaLysX (Asn-Thr) with those of SaArgX (Gly-Phe) leads to substrate preference for glutamate over AAA, contrasting distinctly with the substrate specificity of wild-type SaLysX (11). In the case of NgArgX, the replacement of the two residues (Ala-Leu) in its signature motif by those of SaLysX (Asn-Thr), however, cannot shift the substrate preference of the variant NgArgXa from glutamate to AAA by *in vitro* analysis. Meanwhile, the expression of the NgArgX variants with a SaLysX- or TkLysX-like signature motif cannot endow the Δ*dapB* strain with the ability to biosynthesize lysine from AAA. Moreover, the variants NgArgXe and NgArgXf with the 5-residue signature motif of SaLysX and TkLysX, respectively, are unable to act on not only AAA but also glutamate. It is evident that NgArgX has been evolved to be highly specific for glutamate rather than AAA and is a key determinant of substrate specificity of the ArgW-mediated arginine biosynthetic pathway.

In summary, the endogenous CRISPR-Cas system in strain J7-2 has been harnessed for genome editing and used for functional analysis of the genes involved in arginine and lysine biosynthesis. The results showed that arginine and lysine are produced in strain J7-2 via the ArgW-mediated pathway and the DAP pathway, respectively, and the two pathways are independent of each other. To the best of our knowledge, this is the first report on the arginine biosynthetic pathway mediated by the ArgW/LysW system in haloarchaea. In contrast to the bifunctional LysW-mediated pathways for both lysine and arginine (or ornithine) in hyperthermophilic archaea, the ArgW-mediated pathway in strain J7-2 is a monofunctional pathway for arginine biosynthesis, wherein NgArgX is a key determinant of substrate specificity of this pathway. Given that arginine and lysine biosynthesis enzymes evolved from ancestral enzymes, haloarchaeal enzymes responsible for arginine and lysine biosynthesis have become functionally specialized during evolution.

## MATERIALS AND METHODS

### Strains and growth conditions.

The strains used in this study are listed in Table S1 in the supplemental material. Natrinema gari J7-2 (CCTCC [AB91141]) and its derivatives were grown at 37°C in 18% MGM supplemented with mevinolin (5 μg/mL) when necessary, as described previously (37). The Hv-Min medium (62) was modified and used as the SM for analysis of amino acid requirement of strain J7-2. The SM (per liter) consisted of 3.2 M NaCl, 0.17 M MgSO_4_·7H_2_O, 0.33 M KCl, 5 mM CaCl_2_·2H_2_O, 10 mL of trace elements stock solution, 9.2 mM sodium gluconate, 0.2 mM NH_4_Cl, and 0.6 mM potassium phosphate buffer (pH 7.0). The trace elements stock solution contained (per liter) 5 g EDTA, 0.8 g FeCl_3_, 0.05 g ZnCl_2_, 0.013 g CuCl_2_·2H_2_O, 0.018 g CoCl_2_·6H_2_O, 0.01 g H_3_BO_3_, 2.516 g MnCl_2_·4H_2_O, 0.017 g Ni_2_SO_4_·6H_2_O, and 0.015 g Na_2_MoO_4_·2H_2_O, was adjusted to pH 7.0 with NaOH, filter sterilized, and stored at room temperature (63). E. coli DH5a and E. coli JM110 were used as hosts for plasmid construction and demethylation, respectively, and E. coli BL21(DE3) was used as the expression host; they were grown at 37°C in Luria-Bertani (LB) medium supplemented with ampicillin (100 mg/mL) as needed. Solid medium was prepared by adding 1.5% (wt/vol) agar.

### Plasmid construction.

The plasmids and primers used in this study are listed in Tables S2 and S3, respectively. Plasmids for invader challenging assays were constructed according to the method of Li et al. (43). Briefly, a sticky fragment comprising a designed PAM sequence and a subsequent spacer-matching protospacer was generated by annealing two different-sized primers, and then inserted into the BamHI-XbaI site of the vector pSHS (see Fig. S3 in the supplemental material) (41, 44). Plasmids for genome editing were constructed by inserting a mini-CRISPR preceded by the *sptA* core promoter (41) and a donor DNA into the BamHI-XbaI site of pSHS using the Sangon Biotech Ready-to-use seamless cloning kit (Sangon Biotech, Shanghai, China) according to the manufacturer’s protocol. The promoter-preceded mini-CRISPR was synthesized by PCR using a set of partially overlapping primers (Fig. S4). The upstream (U) and downstream (D) homologous arms of the target sequence were separately amplified from the strain J7-2 genome and connected by overlapping PCR, yielding the donor DNA. In the genome-editing plasmid for introduction of point mutations into the strain J7-2 genome, the donor DNA contained a codon mutation. To construct complementary plasmids, the target gene and the promoter of the gene encoding a high-abundance blue copper domain protein (NJ7G_0566) (52) were separately amplified from the strain J7-2 genome, connected by overlapping PCR, and then inserted into the BamHI-XbaI site of pSHS. The E. coli expression plasmid was constructed by inserting the target gene into the BamHI-NcoI site of pET15b. The sequences of all recombinant plasmids were verified by DNA sequencing.

### Plasmid challenge assay.

The plasmids were demethylated in E. coli JM110 and then transferred into strain J7-2 using the polyethylene glycol (PEG)-mediated transformation method according to the Halohandbook online protocol (https://haloarchaea.com/wp-content/uploads/2018/10/Halohandbook_2009_v7.3mds.pdf). The transformants were plated on 18% MGM agar plates containing 5 μg/mL mevinolin, and the plates were incubated at 37°C for 5 days. Colonies were counted and expressed as CFU.

### Construction and screening of the mutants of *Nnm. gari* J7-2.

After transformation of strain J7-2 with the demethylated genome-editing plasmid, the transformants were plated and grown on 18% MGM agar plates. For screening of the target mutant, randomly selected colonies were cultivated in liquid 18% MGM; cells were recovered by centrifugation and lysed with double-distilled water (ddH_2_O), followed by PCR analysis with external and internal primer pairs of the target gene. For curing of plasmid, the PCR-verified mutant was subjected to 2 to 3 rounds of subculturing (2 days for each round) in liquid 18% MGM, and the absence of the plasmid was confirmed by PCR analysis. Finally, the mutants were validated by DNA sequencing of the PCR product of the target region of the genome.

### Recombinant protein expression and purification.

E. coli BL21(DE3) cells harboring an expression plasmid for NgArgW were cultivated in LB medium at 37°C until the optical density at 600 nm (OD_600_) reached ~0.7. Production of recombinant NgArgW was induced by the addition of 0.4 mM isopropyl-β-d-thiogalactopyranoside (IPTG), and cultivation continued at 37°C for 4 h. Cells were suspended in 20 mM HEPES-NaOH (pH 7.5) containing 0.5 M NaCl and disrupted by sonication on ice. The soluble fraction was recovered by centrifugation at 13,000 × *g* for 10 min at 4°C and then subjected to Ni-NTA affinity chromatography on an Ni^2+^-charged Chelating Sepharose Fast Flow column (GE Healthcare Bio-Sciences) to purify the His-tagged recombinant protein. The Δ*argWX* strain carrying an expression plasmid for NgArgX or its variant NgArgXa was grown at 37°C in 18% MGM. The mid-log-phase cells were recovered by centrifugation and suspended in 20 mM HEPES-NaOH (pH 7.5) containing 3.0 M KCl, followed by sonication on ice. The His-tagged recombinant proteins were purified by Ni-NTA affinity chromatography in the presence of 3.0 M KCl. The concentration of purified samples was determined using the Bradford method with bovine serum albumin as a standard. The amount of target protein in the partially purified sample was further assessed by band intensity measurement following SDS-PAGE.

### SDS-PAGE and immunoblot analyses.

SDS-PAGE was performed with 12% polyacrylamide gel using a Tris-glycine or Tris-tricine buffer system. Protein samples were precipitated with 20% (wt/vol) trichloroacetic acid (TCA) and washed with acetone before being subjected to SDS-PAGE analysis. In some cases, 8 M urea was included in the loading buffer and the gel for urea-SDS-PAGE. The anti-His tag monoclonal antibody (1:10,000) (Novagen) and the alkaline phosphatase (AP)-conjugated goat anti-mouse IgG secondary antibody (1:5,000) (Abbkine, China) were used for immunoblot analysis, and the immunoreactive proteins were visualized using the BCIP/NBT (5-bromo-4-chloro-3-indolylphosphate–nitroblue tetrazolium) chromogen kit (Solarbio, Beijing, China) as described previously (64). In some cases, horseradish peroxidase (HRP)-conjugated goat anti-mouse IgG was used as the secondary antibody (1:5,000) (Sangon Biotech, China), and the signals were detected using Pierce ECL Western blotting substrate (Thermo Fisher Scientific, USA). The images were photographed using the GE Amersham Imager AI680 with an exposure time of 1 or 5 min.

### *In vitro* activity assay for NgArgX and its variant.

The activity of NgArgX or its variant to catalyze the covalent linkage of glutamate or AAA to NgArgW was determined according to the method described by Ouchi et al. (11), with modifications. Briefly, the reaction was carried out at 45°C for 12 h in a mixture containing 10 μg/mL NgArgX or its variant, NgArgW, 10 mM glutamate or AAA, 20 mM ATP, 1 mM MgCl_2_, 0.1 mM ZnSO_4_, and 3 M KCl in 100 mM HEPES (pH 7.5). The reaction mixture was subjected to in-solution digestion by trypsin as described previously (52). The trypsin-digested peptides were determined by nano-LC-MS/MS using an Easy-NanoLC system coupled online with the Q Exactive-HF mass spectrometer (Thermo Scientific, San Jose, CA). The peptide sequences were identified using Proteome Discoverer 2.5 software (Thermo Scientific) to determine the chemical structure of the reaction product.

### Protein structure prediction.

The structural models of NgArgW and NgArgX were predicted by RoseTTAFold (50) or SWISS-MODEL (51). Chimera software (65) was used for visualization of the predicted structure.

### Data availability.

The genome sequence of Natrinema gari J7-2 has been deposited in GenBank under accession no. CP003412.
