# Supplementary material for: Dissecting the Arginine and Lysine Biosynthetic Pathways and Their Relationship in Haloarchaeon Natrinema gari J7-2 via Endogenous CRISPR-Cas System-Based Genome Editing
Source: Microbiol Spectr. 2023 Jun 22;11(4):e00288-23. doi: 10.1128/spectrum.00288-23 (PMC10433800; doi:10.1128/spectrum.00288-23)
Supplement: Supplemental file 1 — Supplemental material. Download spectrum.00288-23-s0001.pdf, PDF file, 1.2 MB [file spectrum.00288-23-s0001.pdf]

SUPPLEMENTAL MATERIAL

Dissecting the arginine and lysine biosynthetic pathways and their relationship in haloarchaeon *Natrinema gari* J7-2 via endogenous CRISPR-Cas system-based genome editing

Yi Wu,[a] Jia Zhang,[b] Bingxue Wang,[a] Yanyan Zhang,[b] Huai Li,[b] Yang Liu,[a] Jing Yin,[a] Dan He,[b] Hongyi Luo,[b] Fei Gan,[a] Bing Tang,[b,c] # Xiao-Feng Tang [a,c] #

[a]Hubei Key Laboratory of Cell Homeostasis, College of Life Sciences, Wuhan University, Wuhan, China

[b]State Key Laboratory of Virology, College of Life Sciences, Wuhan University, Wuhan, China

[c]Cooperative Innovation Center of Industrial Fermentation (Ministry of Education & Hubei Province), Wuhan, China

#Address correspondence to Xiao-Feng Tang, tangxf@whu.edu.cn or Bing Tang, tangb@whu.edu.cn

TABLE S1 Strains used in this study

Strains	Description	Source/reference
***Escherichia coli* strains**		
DH5α	F^- φ80d/lacZΔM15 Δ(*lacZYA-argF*) U169 *deoR recA*1 *hsdR*17(rk - mk^-) *phoA supE*44 λ^- *thi*-1 *gyrA*96 *relA*1	Hanahan et al.,1983
JM110	*dam dcm supE*44 *hsdR*17 *thi leu rpsL1 lacY galK galT ara tonA thr tsx* (*lac-proAB*)/F' [*traD*36 *proAB*$^+$ *lacI q lacZ*ΔM15]	Yanisch-Perron et al., 1985
BL21(DE3)	F^- *omp*T *hsdS$_B$* (r_B^-, m_B^-) *gal dcm* (DE3)	Novagen
***Natrinema gari* strains**		
J7-2	A subculture of *Nnm. gari* J7 (CCTCC AB 91141) lacking the plasmid pHH205	Feng et al., 2012
Δ*crtB*	J7-2 mutant with a deletion of *crtB*	This study
Δ*argWX*	J7-2 mutant with a deletion of both *argW* and *argX*	This study
Δ*argX*	J7-2 mutant with a deletion of *argX*	This study
Δ*argW*	J7-2 mutant with a deletion of *argW*	This study
argW(E54A)	J7-2 mutant with the Glu54 of *argW*-encoded protein being replaced by Ala	This study
Δ*dapB*	J7-2 mutant with a deletion of *dapB*	This study
Δ*argB*	J7-2 mutant with a deletion of *argB*	This study
Δ*argD*	J7-2 mutant with a deletion of *argD*	This study
Δ*hyp4048*	J7-2 mutant with a deletion of *hyp4048*	This study

TABLE S2 Plasmids used in this study

Plasmids	Description	Source/reference
**For plasmid challenge assay**		
pYC-SHSmcs (pSHS)	11.4 kb; *E. coli-Nnm. gari* J7 shuttle vector, Mev^{R}, Amp^{R}. Named as pSHS for short here.	Wang et al. 2016
p1-36	modified pSHS carrying spacer 1-36 preceded by TTC	This study
p1-18	modified pSHS carrying spacer 1-18 preceded by TTC	This study
p1-1	modified pSHS carrying spacer 1-1 preceded by TTC	This study
pAGC	modified pSHS carrying spacer 1-36 preceded by AGC	This study
pCTC	modified pSHS carrying spacer 1-36 preceded by CTC	This study
pTTT	modified pSHS carrying spacer 1-36 preceded by TTT	This study
pCCC	modified pSHS carrying spacer 1-36 preceded by CCC	This study
pTAT	modified pSHS carrying spacer 1-36 preceded by TAT	This study
pTGT	modified pSHS carrying spacer 1-36 preceded by TGT	This study
pGTA	modified pSHS carrying spacer 1-36 preceded by GTA	This study
pATG	modified pSHS carrying spacer 1-36 preceded by ATG	This study
pCGG	modified pSHS carrying spacer 1-36 preceded by CGG	This study
pGAG	modified pSHS carrying spacer 1-36 preceded by GAG	This study
pGAC	modified pSHS carrying spacer 1-36 preceded by GAC	This study
pGCA	modified pSHS carrying spacer 1-36 preceded by GCA	This study
pAGA	modified pSHS carrying spacer 1-36 preceded by AGA	This study
pAAA	modified pSHS carrying spacer 1-36 preceded by AAA	This study
**For mutant construction**		
pKC	pSHS-derived genome-editing vector carrying a 0.8-kb donor DNA and a *crtB*-targeting mini-CRISPR	This study
pKWX	pSHS-derived genome-editing vector carrying a 1-kb donor DNA and an *argWX*-targeting mini-CRISPR	This study
pKX	pSHS-derived genome-editing vector carrying a 0.9-kb donor DNA and an *argX*-targeting mini-CRISPR	This study
pKW	pSHS-derived genome-editing vector carrying a 1-kb donor DNA and an *argW*-targeting mini-CRISPR	This study
pKW-E54A	pSHS-derived genome-editing vector carrying a 1.1-kb donor DNA with the 3'-end CTC mutated to CGC and an *argW*-targeting mini-CRISPR recognizing the PAM sequence CTC	This study

pKDB	pSHS-derived genome-editing vector carrying a 1-kb donor DNA and a *dapB*-targeting mini-CRISPR	This study
pKAB	pSHS-derived genome-editing vector carrying a 1-kb donor DNA and an *argB*-targeting mini-CRISPR	This study
pKAD	pSHS-derived genome-editing vector carrying a 1-kb donor DNA and an *argD*-targeting mini-CRISPR	This study
pK4048	pSHS-derived genome-editing vector carrying a 1-kb donor DNA and a *hyp4048*-targeting mini-CRISPR	This study
**For complementary experiment**		
pSHS-*argWX*	pSHS-derived vector carrying the promoter of *NJ7G_0566* (P_{0566}) and the ORFs of *argW* and *argX* with a C-terminal His tag coding sequence	This study
pSHS-*argX*	pSHS-derived vector carrying P_{0566} preceded ORF of *argX* with a C-terminal His tag coding sequence	This study
pSHS-*argXa*	modified pSHS-*argX* with the mutation A261N/L262T in NgArgX	This study
pSHS-*argXb*	modified pSHS-*argX* with the mutation A261N/L262T/D263V/D264R/A265V/V266N in NgArgX	This study
pSHS-*argXc*	modified pSHS-*argX* with the mutation A261N/L262A in NgArgX	This study
pSHS-*argXd*	modified pSHS-*argX* with the mutation L185F/M195I/A206T/A261N/L262T in NgArgX	This study
pSHS-*argXe*	modified pSHS-*argX* with the mutation L185F/M195I/A206T/A261N/L262T/D263V/D264R/A265V/V266N in NgArgX	This study
pSHS-*argXf*	modified pSHS-*argX* with the mutation L185Y/M195I/A206T/A261N/L262A in NgArgX	This study
pSHS-*argW*	pSHS-derived vector carrying P_{0566} preceded ORF of *argW* with an N-terminal His tag coding sequence	This study
pSHS-*dapB*	pSHS-derived vector carrying P_{0566} preceded ORF of *dapB* with a C-terminal His tag coding sequence	This study
pSHS-*argB*	pSHS-derived vector carrying P_{0566} preceded ORF of *argB* with a C-terminal His tag coding sequence	This study
pSHS-*argD*	pSHS-derived vector carrying P_{0566} preceded ORF of *argD* with a C-terminal His tag coding sequence	This study
**For protein expression in *E. coli* BL21(DE3)**		
pET15b	expression vector in *E. coli*, Cam^R, Amp^R	Novagen
pET15b-*argW*	pET15b-derived expression plasmid for NgArgW with a C-terminal His-tag	This study
pET15b-*argW'*	pET15b-derived expression plasmid for NgArgW with a N-terminal His-tag	This study
pET15b-*argX*	pET15b-derived expression plasmid for NgArgX with a C-terminal His-tag	This study
pET15b-*argXa*	pET15b-derived expression plasmid for NgArgXa with a C-terminal His-tag	This study

TABLE S3 Primers used in this study

Primer	Sequence (5'-3')[a]
**For construction of invader plasmids**	
TTC1-36-F	GATCCTTC**CAGGCGTGGCGCGTTCGCAAGCGAGCTGGGCTCG**T
TTC1-36-R	CTAGA**CGAGCCCAGCTCGCTTGCGAACGCGCCACGCCTG**GAAG
TTC1-18-F	GATCCTTC**GCGTTAGACTCCTCGACGCTGGTAAAAGTCTACGG**T
TTC1-18-R	CTAGA**CCGTAGACTTTTACCAGCGTCGAGGAGTCTAACGC**GAAG
TTC1-1-F	GATCCTTC**CTGGCGCGGTGCGTCGGGAGAACGGTATCGACCTGA**T
TTC1-1-R	CTAGA**TCAGGTCGATACCGTTCTCCCGACGCACCGCGCCAG**GAAG
AGC1-36-F	GATCCAGC**CAGGCGTGGCGCGTTCGCAAGCGAGCTGGGCTCG**T
AGC1-36-R	CTAGA**CGAGCCCAGCTCGCTTGCGAACGCGCCACGCCTG**GCTG
CTC1-36-F	GATCCCTC**CAGGCGTGGCGCGTTCGCAAGCGAGCTGGGCTCG**T
CTC1-36-R	CTAGA**CGAGCCCAGCTCGCTTGCGAACGCGCCACGCCTG**GAGG
TTT1-36-F	GATCCTTT**CAGGCGTGGCGCGTTCGCAAGCGAGCTGGGCTCG**T
TTT1-36-R	CTAGA**CGAGCCCAGCTCGCTTGCGAACGCGCCACGCCTG**AAAG
CCC1-36-F	GATCCCCC**CAGGCGTGGCGCGTTCGCAAGCGAGCTGGGCTCG**T
CCC1-36-R	CTAGA**CGAGCCCAGCTCGCTTGCGAACGCGCCACGCCTG**GGGG
TAT1-36-F	GATCCTAT**CAGGCGTGGCGCGTTCGCAAGCGAGCTGGGCTCG**T
TAT1-36-R	CTAGA**CGAGCCCAGCTCGCTTGCGAACGCGCCACGCCTG**ATAG
TGT1-36-F	GATCCTGT**CAGGCGTGGCGCGTTCGCAAGCGAGCTGGGCTCG**T
TGT1-36-R	CTAGA**CGAGCCCAGCTCGCTTGCGAACGCGCCACGCCTG**ACAG
GTA1-36-F	GATCCGTA**CAGGCGTGGCGCGTTCGCAAGCGAGCTGGGCTCG**T
GTA1-36-R	CTAGA**CGAGCCCAGCTCGCTTGCGAACGCGCCACGCCTG**TACG
ATG1-36-F	GATCCATG**CAGGCGTGGCGCGTTCGCAAGCGAGCTGGGCTCG**T
ATG1-36-R	CTAGA**CGAGCCCAGCTCGCTTGCGAACGCGCCACGCCTG**CATG
CGG1-36-F	GATCCCGG**CAGGCGTGGCGCGTTCGCAAGCGAGCTGGGCTCG**T
CGG1-36-R	CTAGA**CGAGCCCAGCTCGCTTGCGAACGCGCCACGCCTG**CCGG
GAG1-36-F	GATCCGAG**CAGGCGTGGCGCGTTCGCAAGCGAGCTGGGCTCG**T
GAG1-36-R	CTAGA**CGAGCCCAGCTCGCTTGCGAACGCGCCACGCCTG**CTCG
GAC1-36-F	GATCCGAC**CAGGCGTGGCGCGTTCGCAAGCGAGCTGGGCTCG**T

GAC1-36-R	CTAGA**CGAGCCCAGCTCGCTTGCGAACGCGCCACGCCTG**GTCG
GCA1-36-F	GATCCGCA**CAGGCGTGGCGCGTTCGCAAGCGAGCTGGGCTCG**T
GCA1-36-R	CTAGA**CGAGCCCAGCTCGCTTGCGAACGCGCCACGCCTG**TGCG
AGA1-36-F	GATCCAGA**CAGGCGTGGCGCGTTCGCAAGCGAGCTGGGCTCG**T
AGA1-36-R	CTAGA**CGAGCCCAGCTCGCTTGCGAACGCGCCACGCCTG**TCTG
AAA1-36-F	GATCCAAA**CAGGCGTGGCGCGTTCGCAAGCGAGCTGGGCTCG**T
AAA1-36-R	CTAGA**CGAGCCCAGCTCGCTTGCGAACGCGCCACGCCTG**TTTG

For construction of gene-targeting mini CRISPRs [b]

> Universal primers

MC-F	TACAATTGACCGGTTCTAGAATGTTTAGGAAGAATTTAATAGCGTGCTGGAATTTCTTC
MC-R1	*GCTTCGACCCCACAAGGGTCCGTCTGTAAC*AACGCAACCGAAGAAATTCCAGCACGCTA
MC-R4	GCCTGCAGGTCGACTCTAGAAAAAAAAA*GCTTCGACCCCACAAG*

> Specific primers for the *crtB*-targeting CRISPR

crtB-MC-R2	*AAC***GAGGACGTCGCGATCCCCCACGCCGTCACACTCGGC***GCTTCGACCCCACAAGGGTC*
crtB-MC-R3	AAAAAAAA*GCTTCGACCCCACAAGGGTCCGTCTGTAAC***GAGGACGTCGCGATCC**

> Specific primers for the *argWX*-targeting CRISPR

argWX-MC-R2	*AC***CGGACGTCGATCTTCGTGACCTCGTGGTCACGCTCGC***GCTTCGACCCCACAAGGGTC*
argWX-MC-R3	AGAAAAAAAAA*GCTTCGACCCCACAAGGGTCCGTCTGTAAC***CGGACGTCGATCTTCGTG**

> Specific primers for the *argW*-targeting CRISPR

argW-MC-R2	*AC***CGAGTGTGGGGCCGAAGTGTCCCTGCACGACGATTTG***GCTTCGACCCCACAAGGGTC*
argW-MC-R3	AGAAAAAAAAA*GCTTCGACCCCACAAGGGTCCGTCTGTAAC***CGAGTGTGGGGCCGAAGT**

> Specific primers for the *argW*(E54A)-targeting CRISPR

argW(E54A)-MC-R2	*AC***CCTCGATCGAGCCCCCGAGCTCGAAGAGGACTGGGGT***GCTTCGACCCCACAAGGGTC*
argW(E54A)-MC-R3	AGAAAAAAAAA*GCTTCGACCCCACAAGGGTCCGTCTGTAAC***CCTCGATCGAGCCCCCGA**

> Specific primers for the *argX*-targeting CRISPR

argX-MC-R2	*AC***CGGACGTCGATCTTCGTGACCTCGTGGTCACGCTCGC***GCTTCGACCCCACAAGGGTC*
argX-MC-R3	AGAAAAAAAAA*GCTTCGACCCCACAAGGGTCCGTCTGTAAC***CGGACGTCGATCTTCGTG**

> Specific primers for the *argB*-targeting CRISPR

argB-MC-R2	*AC***TGCCCTCGCGGACGTCGCGCGCCTCGTCGAGGACGGC***GCTTCGACCCCACAAGGGTC*

argB-MC-R3	AAAAAAAA*GCTTCGACCCCACAAGGGTCCGTCTGTAAC***TGCCCTCGCGGACGTCGCGC**
**> Specific primers for the *argD*-targeting CRISPR**	
argD-MC-R2	*AC***CACCGCGCTCGAGACCGATCGGCTTGCTCCCGGAAAC***GCTTCGACCCCACAAGGGTC*
argD-MC-R3	AAAAAAAA*GCTTCGACCCCACAAGGGTCCGTCTGTAAC***CACCGCGCTCGAGACCGATC**
**> Specific primers for the *dapB*-targeting CRISPR**	
dapB-MC-R2	*AC***AATCGGCGTCACCGGCGCGACCGGCCGCATGGGTCGG***GCTTCGACCCCACAAGGGTC*
dapB-MC-R3	AAAAAAAA*GCTTCGACCCCACAAGGGTCCGTCTGTAAC***AATCGGCGTCACCGGCGCGA**
**> Specific primers for the *hyp4048*-targeting CRISPR**	
hyp4048-MC-R2	*AC***CGCCGACCGGTAGTTGCGTGACCCAGACCGGTGCAGT***GCTTCGACCCCACAAGGGTC*
hyp4048-MC-R3	AAAAAAAA*GCTTCGACCCCACAAGGGTCCGTCTGTAAC***CGCCGACCGGTAGTTG**
**For construction of donors**	
U1-F	ACCGACTGACGAGGGATCCTCGAACTCGTCAGCTAC
UD1-R	TTGATAAACCGCCGGTCTCTGTCCCGTAGATCCTGCCGACGGTGA
UD1-F	TCTACGGGACAGAGACCGGCGGTTTATCAAAGCCCACGATATAGT
D1-R	GTCAATTGTACGTAGGATCCTAACTCGATCGAGTGCCC
U2-F	ACCGACTGACGAGGGATCCCCTCGAGGCCGCCGCTCA
UD2-R	CGGTCCCGACCGCCATCATGTACTCCCATGTGTGTTCCG
UD2-F	ACACATGGGAGTACATGATGGCGGTCGGGACCGA
D2-R	GTCAATTGTACGTAGGATCCCCCGAGGATCGTCGCGGT
U3-F	ACCGACTGACGAGGGATCCACTACGACGCCCTCGA
UD3-R	TCCTACGTGCAAGGTCACTCTGTACTCCCATGTGTGTTCC
UD3-F	GGAACACACATGGGAGTACAGAGTGACCTTGCACGTAGGA
D3-R	GTCAATTGTACGTAGGATCCGTGCTCGTAGTGGCCA
U4-F	ACCGACTGACGAGGGATCCCGCGCTCGAGACGGCACG
UD4-R	GGTCTCGGTCCCGACCGCCATCAGGTCACTCACCCCAGTCCTC
UD4-F	GAGGACTGGGGTGAGTGACCTGATGGCGGTCGGGACCGAGACC
D4-R	GTCAATTGTACGTAGGATCCCACCCTCGAGCCCGTCGC
U5-F	ACCGACTGACGAGGGATCCCCTCGAGGCCGCCGCTCA
UD5-R	AGTCCTACGTGCAAGGTCACGCACCCCAGTCCTCTTC

UD5-F	GAAGAGGACTGGGGTGCGTGACCTTGCACGTAGGACT
D5-R	GTCAATTGTACGTAGGATCCGTGCTCGTAGTGGCCA
U6-F	ACCGACTGACGAGGGATCCAGGCCTCGAGCCACCCC
UD6-R	CGAGGTCGCTCATTGGTTAGGGTGCCCCCACG
UD6-F	TGGGGGCACCCTAACCAATGAGCGACCTCGATTTCGT
D6-R	GTCAATTGTACGTAGGATCCGGCCGTCGCCGTAGGGG
U7-F	ACCGACTGACGAGGGATCCGAGGACGGTAAGAAGAAGATCAAA
UD7-R	TGGTTGCACTCATGCGCTCATTGGGCCACCTCC
UD7-F	GGTGGCCCAATGAGCGCATGAGTGCAACCATGGAC
D7-R	GTCAATTGTACGTAGGATCCCGTCGCGCCGCTTGGCT
U8-F	ACCGACTGACGAGGGATCCGGCCGGAACATCGAGCC
UD8-R	CGAGTGCGCTCATTCCGTCATCGCTCATCCTCC
UD8-F	GGATGAGCGATGACGGAATGAGCGCACTCGAAACC
D8-R	GTCAATTGTACGTAGGATCCGCCGCCGATGAGCGTGTT
U9-F	ACCGACTGACGAGGGATCCCAGCGCCCGCTTCGTGC
UD9-R	CGTAACCGGTCCGCGCGGATCTCCCTCCTCAAC
UD9-F	GAGGAGGGAGATCCGCGCGGACCGGTTACGCCA
D9-R	GTCAATTGTACGTAGGATCCCATCGGCGGCGTCTTAGA

For PCR screening and DNA sequencing

co-f	CCTGAGCGAGCACGGCGC
co-r	ACTGACGGCAGTTCGGTC
ci-f	CGACGAAGTGGTCGACGA
ci-r	CATCGCCCCCGCGAACGA
argW-U-F	ACCGACTGACGAGGGATCCACTACGACGCCCTCGA
argW-D-R	GTCAATTGTACGTAGGATCCGTGCTCGTAGTGGCCA
argW-I-R	ATGACCGAATGCGTCGAGTG
argX-U-F	ACCGACTGACGAGGGATCCCGCGCTCGAGACGGCACG
argX-D-R	GTCAATTGTACGTAGGATCCCACCCTCGAGCCCGTCGC
argX-I-F	TTGTGCTCGTAGTGGCCAAG

argX-I-R	GTGACCTTGCACGTAGGACT
argWX-U-F	ACCGACTGACGAGGGATCCCCTCGAGGCCGCCGCTCA
argWX-D-R	GTCAATTGTACGTAGGATCCCCCGAGGATCGTCGCGGT
argB-U-F	ACCGACTGACGAGGGATCCAGGCCTCGAGCCACCCC
argB-D-R	GTCAATTGTACGTAGGATCCGGCCGTCGCCGTAGGGG
argB-I-F	ATGACGACCGTCGTGAAGAT
argB-I-R	CCGGATTGCTCCGTTCCGAG
argD-U-F	ACCGACTGACGAGGGATCCGAGGACGGTAAGAAGAAGATCAAA
argD-D-R	GTCAATTGTACGTAGGATCCCGTCGCGCCGCTTGGCT
argD-I-F	GACCTCGATTTCGTTTCCGG
argD-I-R	GATCACGGCCGCGGTCTCGT
dapB-U-F	ACCGACTGACGAGGGATCCGGCCGGAACATCGAGCC
dapB-D-R	GTCAATTGTACGTAGGATCCGCCGCCGATGAGCGTGTT
dapB-I-F	CGGGAAGTGATCGCCGTGGC
dapB-I-R	CCTTCGCGGCCGTGCGTGCG
hyp4048-U-F	ACCGACTGACGAGGGATCCCAGCGCCCGCTTCGTGC
hyp4048-D-R	GTCAATTGTACGTAGGATCCCATCGGCGGCGTCTTAGA
hyp4048-I-F	CACTGCACCGGTCTGGGT
hyp4048-I-R	GGATCGTCGTTTTCGGGA
**For monitoring the plasmid curing**	
s1-f	TACCCGAATCAAATGGGAGT
s1-r	GCATTGTGCGGATCTTGCTT
**For construction of complementary plasmids**	
P0566-F1	TACAATTGACCGGTTCTAGAGGCAGCCTCGGCTTC
argW-NH-R1	TCGACGCATTCGGTATGATGATGATGATGATGCATCACCGGGGTTTGGGCCTGAC
argW-F2	CATCATACCGAATGCGTCGA
argW-R2	GACCATGATTACGCCAAGCTTAAAAAAAATCACTCACCCCAGTCCTC
argX-R1	AGTCCTACGTGCAAGGTCACCACCGGGGTTTGGGC

argX-F2	GTGACCTTGCACGTAGGACT
argX-R2	CCAAGCTTAAAAAAAATCAGTGGTGGTGGTGGTGGTGGGCGGTCACCTCGAGTTCGT
T8-R3	ATGACCATGATTACGCCAAGCTTAAAAAAAA
*argX*a-R	GACGGCGTCGTCTGTGTTCTTGAACTCGACGGT
*argX*a-F	GTCGAGTTCAAGAACACAGACGACGCCGTCGAG
*argX*b-R	TCTCGTTGACGCGGACTGTGTTCTTGAACTCGACGGT
*argX*b-F	AAGAACACAGTCCGCGTCAACGAGACGGACATCGCG
*argX*c-R	GACGGCGTCGTCCGCGTTCTTGAACTCGACGGT
*argX*c-F	GTCGAGTTCAAGAACGCGGACGACGCCGTCGAG
*argX*d-R	GACGAGCGGACAATCGCGGCGATGGGTTCGCCGTCGGTCGCAAACACGCGGATGTCACG
*argX*d-F	TCGCCGCGATTGTCCGCTCGTCGGACCACTGGATCACCAACACAGCGAAGGGTGCGGAG
*argX*e-R	GACGAGCGGACAATCGCGGCGATGGGTTCGCCGTCGGTCGCAAACACGCGGATGTCACG
*argX*e-F	TCGCCGCGATTGTCCGCTCGTCGGACCACTGGATCACCAACACAGCGAAGGGTGCGGAG
*argX*f-R	GACGAGCGGACAATCGCGGCGATGGGTTCGCCGTCGGTCGCGTACACGCGGATGTCACG
*argX*f-F	TCGCCGCGATTGTCCGCTCGTCGGACCACTGGATCACCAACACAGCGAAGGGTGCGGAG
argWX-R1	CACTCGACGCATTCGGTCATCACCGGGGTTTGGGC
argWX-F2	ATGACCGAATGCGTCGAGTG
argB-R1	ATCTTCACGACGGTCGTCATCACCGGGGTTTGGGC
argB-F2	ATGACGACCGTCGTGAAGAT
argB-R2	CCAAGCTTAAAAAAAATCAGTGGTGGTGGTGGTGGTGTTGGGCCACCTCCTCGCCGT
argD-R1	ATCGAGGTCGCTCATCACCGGGGTTTGGGCCTG
argD-F2	GCCCAAACCCCGGTGATGAGCGACCTCGATTTC
argD-R2	CCAAGCTTAAAAAAAATCAGTGGTGGTGGTGGTGGTGTGCGTTGGAGTCGGTCTCGG
dapB-R1	ACGCCGATTCGCGTCGTCATCACCGGGGTTTGGGC
dapB-F2	ATGACGACGCGAATCGGCGT
dapB-R2	CAAGCTTAAAAAAAATCAGTGGTGGTGGTGGTGGTGTTCGCTGATCACGTCTGCGA

For construction of expression plasmid to produce recombinant protein in *E. coli*

argW-F	AAGAAGGAGATATACCATGGGCACCGAATGCGTCGAGTGTGG
argW-CH-R	CTTTGTTAGCAGCCGGATCCTCAGTGGTGGTGGTGGTGGTGCTCACCCCAGTCCTCTTC

argW-NH-F	AAGAAGGAGATATACCATGGGCCACCACCACCACCACCACACCGAATGCGTCGAGTGTGG
argW-R	GCTTTGTTAGCAGCCGGATCCTCACTCACCCCAGTCCTCTT
argX-F	AAGAAGGAGATATACCATGGCGTGACCTTGCACGTAGGACT
argX-CH-R	CTTTGTTAGCAGCCGGATCCTCAGTGGTGGTGGTGGTGGTGGGCGGTCACCTCGAGTTC
argXa-F	AAGAAGGAGATATACCATGGCGTGACCTTGCACGTAGGACT
argXa-CH-R	CTTTGTTAGCAGCCGGATCCTCAGTGGTGGTGGTGGTGGTGGGCGGTCACCTCGAGTTC

[a] Restriction sites are underlined. The PAM sequences are shown in blue. The mutated nucleotides are shown in red. The His6 tag-coding DNA sequences are shown in green. The transcription terminators are shown in orange. The spacers or protospacers are in boldface. The italicized sections indicate the repeats.

[b] The universal primers were used repeatedly for constructing different mini-CRISPRs, and the specific primers were used to construct corresponding mini CRISPRs.

TEBLE S4 Transformation efficiencies of *Natrinema gari* J7-2 by pSHS and invader plasmids[#]

Plasmid	Log_{10} CFU/μg DNA			Mean ± SD	*P* value
	Test 1	Test 2	Test 3		
pSHS	3.623	4.790	4.161	4.191 ± 0.477	
p1-36	1.756	1.623	1.699	1.693 ± 0.054	0.000905
p1-18	2.068	2.057	2.083	2.069 ± 0.011	0.001627
p1-1	1.380	1.643	1.491	1.505 ± 0.108	0.000738
pAGC	3.964	3.934	3.914	3.937 ± 0.021	0.246657
pCTC	1.799	1.806	1.785	1.797 ± 0.009	0.001037
pTTT	1.398	1.447	1.322	1.389 ± 0.051	0.000584
pCCC	3.303	3.336	3.326	3.322 ± 0.014	0.030711
pTAT	3.524	3.587	3.450	3.520 ± 0.056	0.059531
pTGT	3.949	3.954	3.919	3.941 ± 0.016	0.249413
pGTA	3.322	3.352	3.286	3.320 ± 0.027	0.030619
pATG	3.279	3.320	3.258	3.286 ± 0.026	0.027503
pCGG	3.318	3.356	3.260	3.311 ± 0.039	0.029954
pGAG	3.591	3.544	3.643	3.593 ± 0.041	0.075761
pGAC	3.934	3.968	3.929	3.944 ± 0.017	0.252049
pGCA	3.813	3.845	3.898	3.852 ± 0.035	0.185942
pAGA	3.258	3.230	3.223	3.237 ± 0.015	0.023658
pAAA	3.924	3.949	3.919	3.931 ± 0.013	0.241439

[#] The data listed in this table were used for preparation of Figs. 2C and 2D.

TEBLE S5 Transformation efficiencies of *Natrinema gari* J7-2 by pSHS and pCK[#]

Plasmid	CFU/μg DNA			Mean ± SD (%)	*P* value
	Test 1	Test 2	Test 3		
pSHS	4100	5900	4700	100 ± 15.261	
pCK	870	540	462	12.733 ± 3.642	0.000706

[#] The data listed in this table were used for preparation of Fig. 3B.

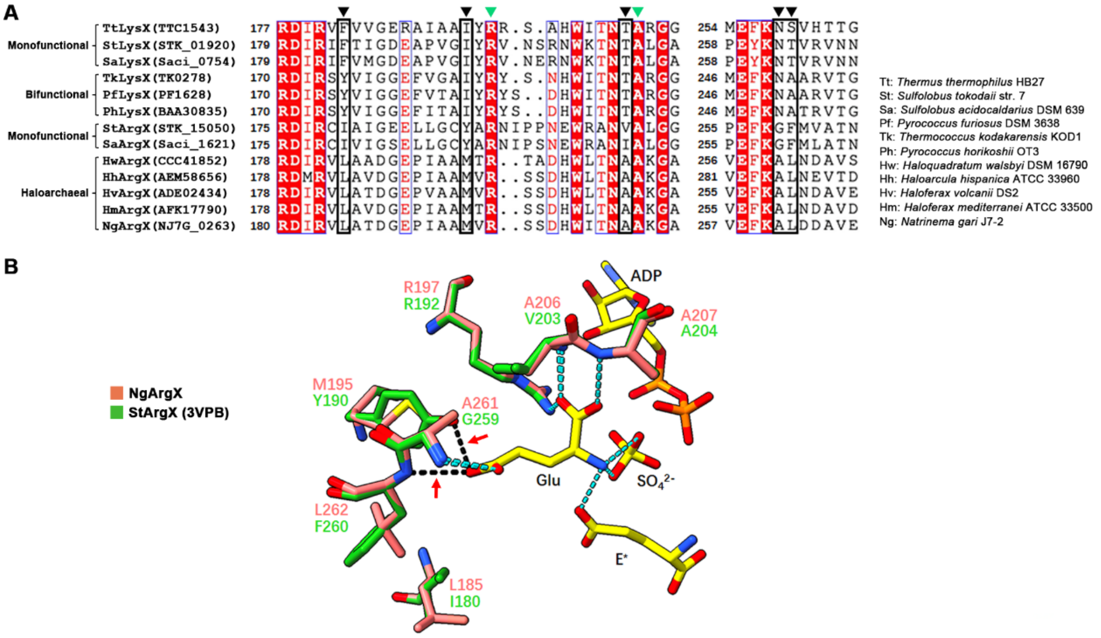

FIG S1 Substrate recognition by NgArgX. (A) Alignment of amino acid sequences around the substrate recognition residues of LysX/ArgX proteins. The five-amino-acid signature residues are indicated by black arrowheads, and the two highly conserved residues involved in substrate recognition are indicated by green arrowheads. GenBank accession numbers of the proteins are shown in parentheses. (B) Superimposition of the glutamate recognition region of the structure model of NgArgX with that of the crystal structure of the LysW-ADP-glutamate-bound complex of StArgX from *S. tokodaii*. The structure model of NgArgX was predicted by SWISS-MODEL with the crystal structure of the LysW-ADP-glutamate-bound complex of StArgX (3VPB) as the template. The red arrows indicate the two hydrogen bonds presented in the complex of StArgX but not in that of NgArgX. "E*" represents the C-terminal Glu residue of LysW/ArgW protein.

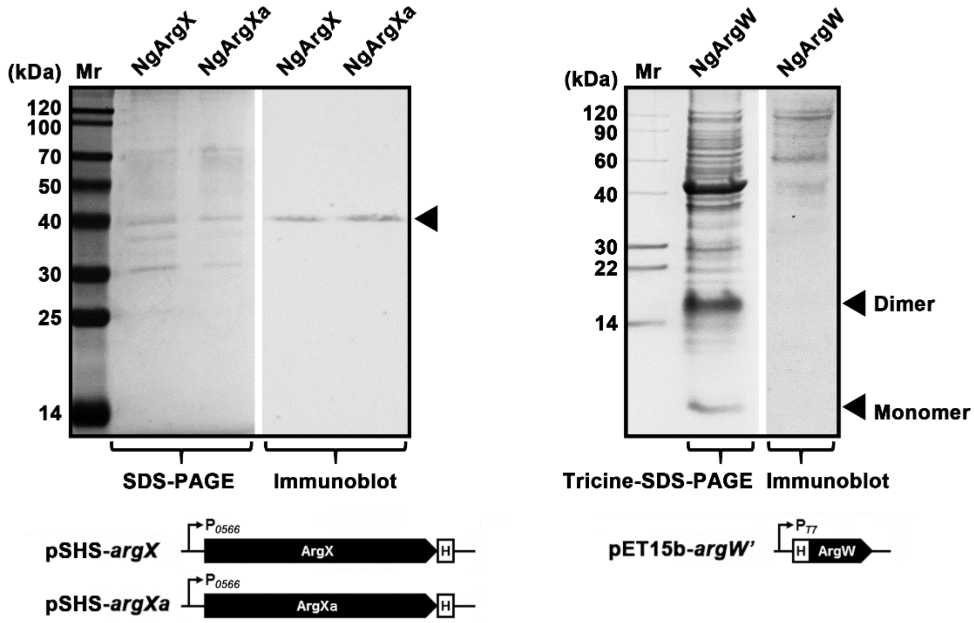

FIG S2 SDS-PAGE and immunoblot analyses of purified samples of recombinant NgArgW with an N-terminal His-tag produced in *E. coli* and NgArgX and NgArgXa with a C-terminal His-tag produced in Δ*argWX*. After purification by Ni-NTA affinity chromatography, the sample was subjected to SDS-PAGE or Tricine-SDS-PAGE as indicated, followed by anti-His-tag immunoblot analysis. Schematic representations of recombinant genes with His-tag (H) coding sequence on expression plasmids are shown. Note: similar to the case of NgArgW with a C-terminal His-tag (Fig. 5F), NgArgW with an N-terminal His-tag also forms dimeric and higher AMW forms, and the monomeric and dimeric forms are hardly detected by anti-His-tag immunoblot analysis.

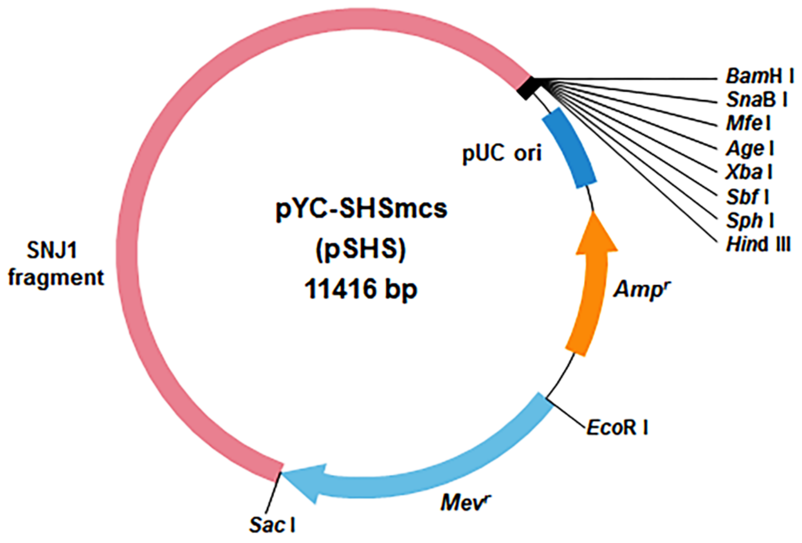

FIG S3 A restriction map of the vector pYC-SHSmcs (named pSHS for short in this study). The SNJ1 fragment contains the replicon region of a temperate haloarchaeal virus (SNJ1) identified in *Natrinema gari* J7 (Wang et al., 2016).

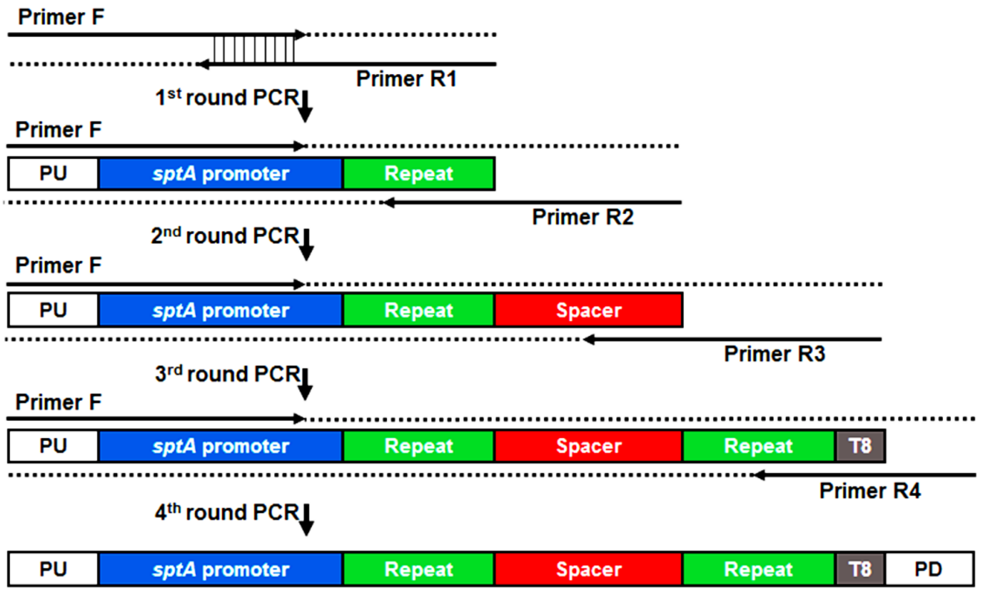

FIG S4 Schematic representation of the construction of mini-CRISPR. The *sptA* promoter-preceded mini-CRISPR containing two repeats and a spacer was synthesized by four rounds of PCR using a set of partially overlapping primers. PU and PD are the sequences identical to corresponding regions of the vector pSHS, and used for inserting the mini-CRISPR into the vector by seamless cloning method. T8 (8 thymidines) is the transcription terminator (Gong et al., 2019).

REFERENCES

Feng J, Liu B, Zhang Z, Ren Y, Li Y, Gan F, Huang Y, Chen X, Shen P, Wang L, Tang B, Tang XF. 2012. The complete genome sequence of *Natrinema* sp. J7-2, a haloarchaeon capable of growth on synthetic media without amino acid supplements. PLoS One 7:e41621.

Gong L, Li M, Cheng F, Zhao, D, Chen Y, Xiang H. 2019. Primed adaptation tolerates extensive structural and size variations of the CRISPR RNA guide in *Haloarcula hispanica*. Nucleic Acids Res 47:5880-5891.

Hanahan D. 1983. Studies on transformation of *Escherichia coli* with plasmids. J Mol Biol 166:557-580.

Wang Y, Sima L, Lv J, Huang S, Liu Y, Wang J, Krupovic M, Chen X. 2016. Identification, characterization, and application of the replicon region of the halophilic temperate sphaerolipovirus SNJ1. J Bacteriol 198:1952-1964.

Yanisch-Perron C, Vieira J, Messing J. 1985. Improved M13 phage cloning vectors and host strains: nucleotide sequences of the M13mp18 and pUC19 vectors. Gene 33:103-119.
